# Immunomodulating nano-adaptors potentiate antibody-based cancer immunotherapy

**DOI:** 10.1038/s41467-021-21497-6

**Published:** 2021-03-01

**Authors:** Cheng-Tao Jiang, Kai-Ge Chen, An Liu, Hua Huang, Ya-Nan Fan, Dong-Kun Zhao, Qian-Ni Ye, Hou-Bing Zhang, Cong-Fei Xu, Song Shen, Meng-Hua Xiong, Jin-Zhi Du, Xian-Zhu Yang, Jun Wang

**Affiliations:** 1 School of Biomedical Sciences and Engineering, South China University of Technology, Guangzhou International Campus, Guangzhou, 511442 PR China; 2 School of Life Sciences, University of Science and Technology of China, Hefei, 230027 PR China; 3 Shenzhen Bay Laboratory, Shenzhen, 518132 PR China; 4 National Engineering Research Center for Tissue Restoration and Reconstruction, South China University of Technology, Guangzhou, 510006 PR China; 5 Key Laboratory of Biomedical Engineering of Guangdong Province, and Innovation Center for Tissue Restoration and Reconstruction, South China University of Technology, Guangzhou, 510006 PR China; 6 Bioland Laboratory (Guangzhou Regenerative Medicine and Health Guangdong Laboratory), Guangzhou, 510005 PR China; 7 Key Laboratory of Biomedical Materials and Engineering of the Ministry of Education, South China University of Technology, Guangzhou, 510006 PR China

**Keywords:** Drug delivery, Nanotechnology in cancer

## Abstract

Modulating effector immune cells via monoclonal antibodies (mAbs) and facilitating the co-engagement of T cells and tumor cells via chimeric antigen receptor- T cells or bispecific T cell-engaging antibodies are two typical cancer immunotherapy approaches. We speculated that immobilizing two types of mAbs against effector cells and tumor cells on a single nanoparticle could integrate the functions of these two approaches, as the engineered formulation (immunomodulating nano-adaptor, imNA) could potentially associate with both cells and bridge them together like an ‘adaptor’ while maintaining the immunomodulatory properties of the parental mAbs. However, existing mAbs-immobilization strategies mainly rely on a chemical reaction, a process that is rough and difficult to control. Here, we build up a versatile antibody immobilization platform by conjugating anti-IgG (Fc specific) antibody (αFc) onto the nanoparticle surface (αFc-NP), and confirm that αFc-NP could conveniently and efficiently immobilize two types of mAbs through Fc-specific noncovalent interactions to form imNAs. Finally, we validate the superiority of imNAs over the mixture of parental mAbs in T cell-, natural killer cell- and macrophage-mediated antitumor immune responses in multiple murine tumor models.

## Abstract (web summary)

Current strategies to boost anti-tumor immune response include the use of immune checkpoint inhibitors and bispecific T cell-engaging antibodies. Here the authors describe a versatile antibody immobilization nanoplatform that can be used to deliver different combinations of immunotherapeutics, showing therapeutic superiority in pre-clinical models.

## Introduction

Current strategies to boost cancer immunotherapy in the clinic include two broad categories. One category is to utilize immunomodulatory monoclonal antibodies (mAbs) to reinvigorate dysfunctional T lymphocytes^1,2^. mAbs that block cytotoxic T-lymphocyte-associated protein 4 (CTLA4), programmed cell death protein 1 (PD1), and programmed cell death ligand 1 (PDL1) represent the predominant modalities for cancer immunotherapy and have revolutionized the cancer treatment paradigm in recent years^3,4^. Of note, numerous mAbs capable of potentiating the antitumor activities of innate immune cells (for example, natural killer cells and macrophages) are also under intense investigation^5–7^.

Parallel to the immunomodulatory mAbs, the other strategy is to facilitate the engagement of effector immune cells and tumor cells via chimeric antigen receptor (CAR) T cells or bispecific T cell-engaging antibodies (BiTEs)^8–10^. The former are engineered autologous T cells expressing an artificial receptor that recognizes a specific tumor-associated antigen (TAA)^11,12^, and the latter is an advanced format of bispecific antibodies (bsAbs) that comprise two-variable fragments capable of simultaneously binding to the T cell receptor complex and TAA^13–15^. Although the anticancer mechanisms are different, CAR T cells and BiTE share a common feature of triggering physical connections between effector cells and tumor cells and facilitating cytotoxicity against the latter^9,16,17^.

To fully exploit the advantages of these two strategies, the combination of immunomodulatory mAbs and CAR T cells or BiTEs have been combined and have shown improved efficacy in comparison with individual approaches alone;^18–20^ however, the two therapeutics in most cases work independently. We speculated that integrating the features of these two strategies into one system may further boost immunotherapy. We proposed that nanoparticle immobilizing two types of mAbs targeting effector cells and tumor cells could be such a system, as the engineered formulation potentially has two distinctive features and acts as an immunomodulating nano-adaptors (imNAs). First, the immobilized parental mAbs maintain their immunomodulatory properties, and second, because of their multivalence, they have a strong affinity for antigens on both cells and can physically bridge them together like an ‘adaptor’^21^. It is reasonable to predict that imNAs could achieve amplified and unachievable antitumor effects over a mixture of two types of mAbs. For the immobilization of mAbs onto nanoparticles, previously reported approaches mainly rely on chemical reactions;^21,22^ however, this process is difficult to control due to the high molecular weight of mAbs and nanoparticles, and it may also hurt the valency of mAbs, limiting their clinical translation^23^.

**Fig. 1:**
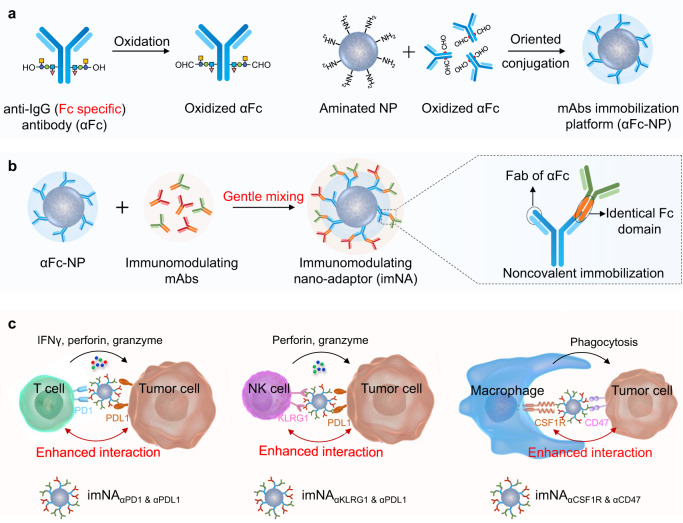
Schematic illustrating the design of imNAs and their potential to improve antibody-based cancer immunotherapy. **a** An anti-IgG (Fc specific) antibody (αFc) was oxidized and oriented conjugated onto the surface of nanoparticle to form an antibody immobilization platform (αFc-NP). **b** Two types of immunomodulating monoclonal antibodies (mAbs) that target effector cells and tumor cells could be immobilized onto αFc-NP after gentle mixing via Fc recognition. The constructed formulations could serve as immunomodulating nano-adaptors (imNAs) as they potentially associate with effector cells and tumor cells simultaneously and bridge them together like an ‘adaptor’ while maintaining the immunomodulatory properties of the parental mAbs. **c** The versatility of αFc-NP and superiority of imNAs were validated in T cell-, natural killer cell- and macrophage-mediated antitumor immune responses in multiple murine tumor models.

In this work, considering that Fc regions are identical or conserved in all IgG antibodies and that an anti-IgG (Fc specific) antibody (αFc) can specifically recognize and bind any mAbs comprising the Fc fragment through noncovalent interactions^24,25^, we propose to build up a versatile antibody immobilization platform by conjugating αFc onto the nanoparticle surface (αFc-NP) (Fig. 1a). We confirm that αFc-NP could conveniently and efficiently immobilize two types of mAbs after gentle mixing to form imNAs without compromising the antigen-binding capabilities of the parental mAbs (Fig. 1b). We further select the immune checkpoint inhibitors αPD1 (an anti-PD1 antibody) and αPDL1 (an anti-PDL1 antibody) as model mAbs and show that imNA_αPD1 & αPDL1_ could effectively promote T cell/tumor cell interactions and strikingly augment T cell-mediated antitumor immunity in vitro and in vivo, in compared with the combination of soluble or nanoparticle-immobilized αPD1 and αPDL1. Notably, the superiority of imNAs over the mixture of parental mAbs is validated in natural killer cell- and macrophage-mediated antitumor immune responses in multiple murine tumor models (Fig. 1c). Collectively, we provide a convenient and well-controlled methodology for the engineering of imNAs, and the simplicity, versatility, and effectiveness of the methodology make imNAs an attractive candidate for clinical translation.

## Results

### Design and characterization of the antibody immobilization platform

**Fig. 2:**
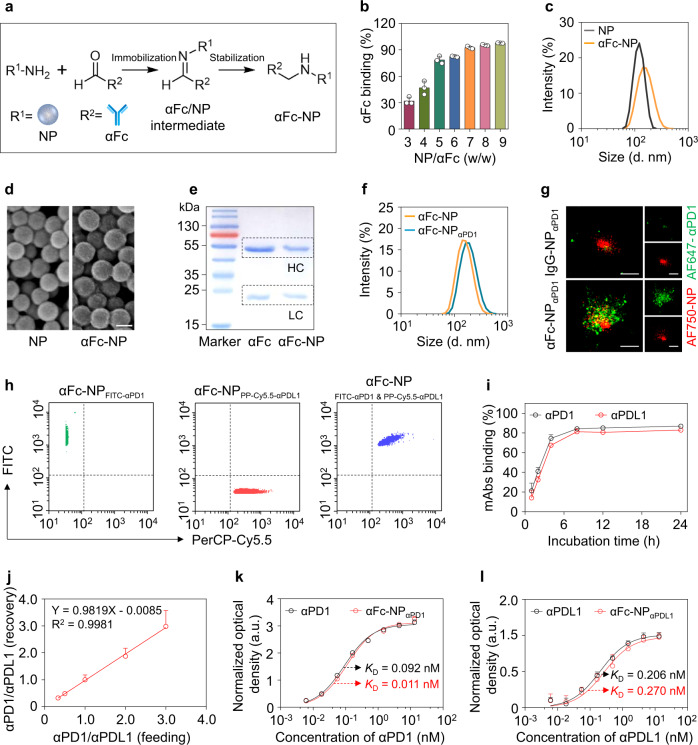
Construction and characterization of the antibody immobilization platform (αFc-NP). **a** Schematic depicting the construction of αFc-conjugated nanoparticles (αFc-NP). αFc was oxidized and immobilized onto aminated polystyrene NP via aldol condensation. **b** Determination of the αFc binding efficacy by ELISA. **c**, Average hydrodynamic size of NP and αFc-NP as determined by DLS, confirming that NP had an average diameter of 123.4 ± 4.1 nm and αFc-NP had an average diameter of 152.8 ± 2.0 nm. **d** Representative scanning electron microscopy (SEM) image of NP and αFc-NP. Scale bar, 100 nm. **e** Reducing SDS-PAGE gel stained with Coomassie Brilliant Blue showing the heavy chain (HC) and light chain (LC) released from soluble αFc or αFc-NP. β-mercaptoethanol treatment breaks the interchain disulfide bonds and separates HC and LC of αFc. The molecular weights of HC and LC are approximately 50 kDa and 25 kDa, respectively. **f** Size distribution of αFc-NP and αFc-NP_αPD1_ determined by DLS, confirming the αFc-NP_αPD1_ had an average diameter of 176.1 ± 5.3 nm, 30 nm larger than αFc-NP. **g** Stochastic optical reconstruction microscopy (STORM) images of IgG-NP_αPD1_ and αFc-NP_αPD1_. NP and αPD1 were labeled with AF750 and AF647, respectively. An NP conjugating IgG control antibody (IgG-NP) was used as a control. Scale bar, 200 nm. **h** Nanoflow Cytometry showed that αFc-NP could simultaneously bind two types of mAbs. αFc-NP was incubated with FITC-labeled αPD1 and PerCP-Cy5.5-labeled αPDL1 separately or in combination. **i** Binding efficacies of αPD1 and αPDL1 versus incubation time. αFc-NP were incubated with αPD1 and αPD1 at an αFc: αPD1: αPDL1 ratio of 1:0.5:0.5 for different periods, and then the unbound αPD1 and αPD1 were then examined by ELISA. **j** The ratio of αPD1 and αPDL1 immobilized by αFc-NP. αFc-NP was incubated with αPD1 and αPDL1 at an αFc: (αPD1 & αPDL1) ratio of 1:1, while the ratio of αPD1 and αPDL1 ranged from 0.3 to 3.0. **k**, **l** The capabilities of αFc-NP_αPD1_ and αFc-NP_αPDL1_ to bind corresponding antigens. Data are the means ± s.d of three different experiments with similar results. Source data are provided as a Source Data file.

To fabricate imNAs conveniently and efficiently, we proposed to build up a versatile antibody immobilization platform by conjugating anti-IgG (Fc specific) antibody (αFc) onto the surface of nanoparticles (NP). Since the Fc regions are identical in all IgG antibodies from the same host species and are well-conserved between different host species^26^, it is reasonable to predict that αFc-conjugated nanoparticles (αFc-NP) could recognize and immobilize any mAb containing the Fc fragment. To maximize the mAb-binding capability of αFc, an oriented conjugation approach was employed to engineer αFc-NP (Fig. 2a): the hydroxyl groups in carbohydrate residues, which are typically located in the heavy chain CH2 domain of αFc^27^, were first oxidized to aldehyde groups by sodium periodate (Supplementary Fig. 1), followed by a condensation reaction with aminated polystyrene NP and reductive amination of Schiff bases with sodium borohydride (Fig. 2a). Over 80% of αFc was immobilized onto NP when the mass ratio of NP to αFc was equal to or greater than 5:1, as determined by enzyme-linked immunosorbent assay (ELISA) (Fig. 2b) and ultra-performance liquid chromatography (UPLC) (Supplementary Fig. 2). Considering the αFc-binding efficacy and αFc-loading content, a NP: αFc ratio of 5:1 was selected for the construction of αFc-NP in the subsequent investigation, and 1 mg NP could bind approximately 160 μg αFc at this ratio, as determined by ELISA.

As measured by dynamic light scattering (DLS), the average hydrodynamic diameter of αFc-NP was ~152.8 nm, approximately 30 nm larger than that of polystyrene NP (Fig. 2c), while the typical size of an antibody molecule is 10-15 nm^28^. Scanning electron microscopy (SEM) images confirmed that both NP and αFc-NP were homogeneously spherical; intriguingly, the former had a smooth surface while the latter had a rough surface (Fig. 2d). Collectively, compared with naked polystyrene NP, αFc-NP showed remarkable changes in terms of size distribution and morphology, indicating that a layer of antibodies was attached to the surface of the NP. To further substantiate that αFc was oriented chemically conjugated but not physically adsorbed onto NP, the heavy chains (HC) and light chains (LC) of soluble αFc and αFc-NP were separated by β-mercaptoethanol, which can break the interchain disulfide bonds, followed by gradient SDS-PAGE (sodium dodecyl sulfate-polyacrylamide gel electrophoresis) and Coomassie Blue staining (Supplementary Fig. 3). As shown in Fig. 2e, in comparison with free αFc counterpart, the HC band (~50 kDa) was substantially dimmer in the αFc-NP group when the LC bands (~25 kDa) were identical; the missing HC was thought to be linked to the NP through carbohydrates and blocked in the sample well during SDS-PAGE process, confirming that αFc was chemically conjugated.

Having validated the successful construction of αFc-NP, we further examined whether αFc-NP could serve as a versatile platform for the immobilization of mAbs containing Fc fragments (Fig. 1c). DLS measurements showed that the average hydrodynamic diameter of αFc-NP increased by approximately 30 nm after incubation with αPD1 at 4 °C for 2 h, indicating the immobilization of αPD1 to αFc-NP (αFc-NP_αPD1_) (Fig. 2f). To verify that αFc-NP_αPD1_ was generated through the specific interaction between αFc and the Fc fragment of αPD1, αFc or an IgG control antibody (anti-trinitrophenol antibody) was conjugated to an Alexa Fluor® 750 (AF750)-labeled NP and then incubated with Alexa Fluor® 647 (AF647)-labeled αPD1, followed by imaging of stochastic optical reconstruction microscopy (STORM) imaging. As shown in Fig. 2g, green fluorescence (AF647) was rarely observed surrounding NP in the IgG-NP_αPD1_ group (upper), while larger formulation and colocalization of two kinds of fluorescence (green and red) were observed in the αFc-NP_αPD1_ group (lower), indicating the integration of αFc-NP and αPD1. Additionally, αFc-NP_αPD1_ remained stable within two days in 5% glucose solution as measured by the variation of the size distribution (Supplementary Fig. 4). More importantly, when incubated with IgG antibodies from the same or other species, few αFc-NP-integrated αPD1 was rarely replaced by surrounding antibodies (Supplementary Fig. 5), even though the concentration of surrounding antibodies was much higher than αPD1 (Supplementary Fig. 6), indicating that the interaction between Fc fragments and αFc was relatively stable.

Next, to examine whether αFc-NP can immobilize more than one type of mAb, αFc-NP was incubated with FITC-labeled αPD1 and PerCP-Cy5.5-labeled αPDL1 separately or in combination and then subjected to nanoflow cytometry. The emergence of FITC^+^PerCP-Cy5.5^+^ formulations in the combination group confirmed that αFc-NP could simultaneously immobilize two types of mAbs, and it could therefore be a universal platform for antibody-based combination therapy (Fig. 2h). Additionally, after gentle mixing and a short incubation (less than 4 h), more than 80% of αPD1 and αPDL1 was incorporated onto αFc-NP at the αFc: αPD1: αPDL1 ratio of 1:0.5:0.5 (Fig. 2i).

As the amount and distribution of antigens on effector cells and tumor cells were different, it was necessary to consider the ratio of the two types of mAbs when used in combination. Intriguingly, we could achieve predetermined ratios of αPD1/αPDL1 by modifying their feeding amounts (Fig. 2j), confirming that αFc-NP can immobilize mAbs in a well-controlled manner. As random covalent immobilization can reduce the antibody’s affinity^29,30^, it is reasonable to speculate that the elaborate strategy we developed could maximally preserve the function of the parental mAbs, which were immobilized through noncovalent interactions but not chemical conjugation. To test this hypothesis, we incubated free αPD1 or αFc-NP_αPD1_ with the recombinant murine PD1 protein and found that attachment to αFc-NP did not affect αPD1’s ability to bind the antigen compared with its free counterpart, as demonstrated by similar dissociation constants (K_D_) (Fig. 2k). αFc-NP_αPDL1_ and free αPDL1 also exhibited similar antigen-binding capabilities (Fig. 2l). Collectively, αFc-NP could serve as a versatile antibody immobilization platform by integrating different types of immunomodulatory mAbs conveniently and efficiently, without impairing their antigen-binding capability.

### imNA_αPD1 & αPDL1_ enhance T cell-mediated cytotoxicity in vitro

**Fig. 3:**
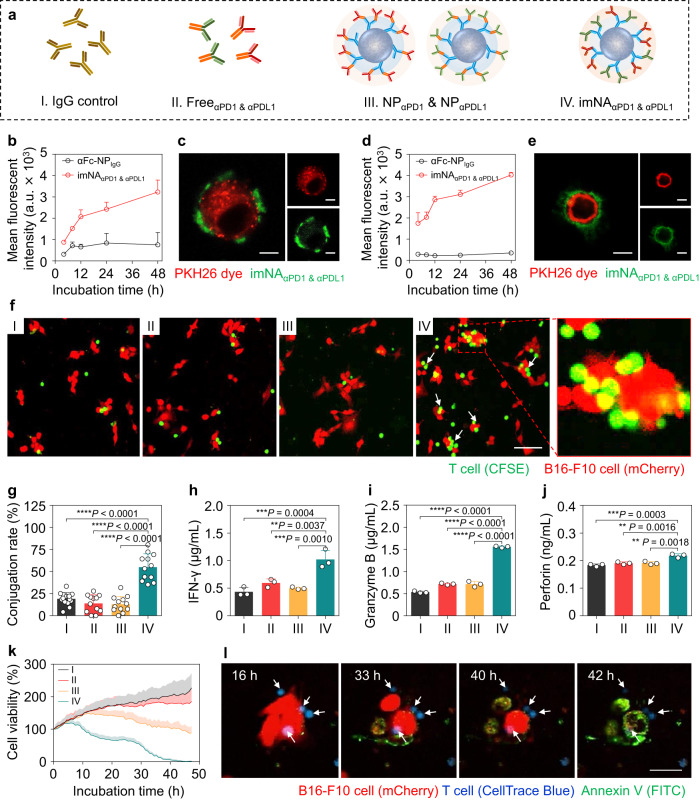
imNA_αPD1 & αPDL1_ increases CD8^+^ T cell-mediated cytotoxicity in vitro. **a** CD8^+^ T cell-mediated antitumor immunity was selected as a model to investigate the superiority of imNA; the following four groups were established: (1) IgG control; (2) Free_αPD1 & αPDL1_; (3) NP_αPD1_ & NP_αPDL1_; and (4) imNA_αPD1 & αPDL1_. **b** Extracellular fluorescence of B16-F10 cells after treatment with αFc-NP_IgG_ or imNA_αPD1 & αPDL1_ for different periods. NPs were labeled with FITC. a.u., arbitrary unit. Data were presented as mean ± s.d. *n* = 3 biologically independent samples. **c** Confocal images of B16-F10 cells showed cell associations with imNA_αPD1 & αPDL1_ after a 24 h incubation. The cell membrane was stained with PKH26 dye. Scale bar, 5 μm. **d** Extracellular fluorescence of CD8^+^ T cells after treatment with αFc-NP_IgG_ or imNA_αPD1 & αPDL1_ for different periods. Data were presented as mean ± s.d. *n* = 3 biologically independent samples. **e** Confocal images of CD8^+^ T cells showing cell association with imNA_αPD1 & αPDL1_ after a 24 h incubation. Scale bar, 5 μm. **f** Confocal images showing the conjugations of CD8^+^ T cells on B16-F10 tumor cells. CFSE-labeled CD8^+^ T cells and B16-F10-mCherry cells were co-incubated for 8 h in the presence of different treatments, and cell conjugations were detected after washing. Scale bar, 50 μm. **g** The conjugation rates were determined manually by observing the red/green fluorescence overlap in multiple nonoverlapping images. Data were presented as mean ± s.d. *n* = 12 nonoverlapping images. The concentrations of IFN-γ (**h**), Granzyme B (**i**), and Perforin (**j**) in the supernatant of coincubated cells were examined by ELISA. CD8^+^ T cells and B16-F10 cells (10:1) were coincubated and treated as indicated above for 24 h. Data are presented as means ± s.d. *n* = 3 biologically independent samples. **k** Viability of B16-F10 cells analyzed by a high content analysis (HCA) platform. **l** HCA images showing that imNA_αPD1 & αPDL1_-facilitated cell interactions induced tumor cells apoptosis. Scale bar, 10 μm. Data are presented as means ± s.d. Statistical significance was calculated via one-way ANOVA with the Tukey post-hoc test. *P < 0.05; **P < 0.01; ***P < 0.001; ****P < 0.0001. Source data are provided as a Source Data file.

We have successfully constructed a versatile antibody immobilization platform, and we predicted that αFc-NP integrating two types of mAbs that targeted effector cells and tumor cells could serve as imNA, as they retained the basic function of the parental mAbs and could also engage the antigens on the surface of both cells and bridge them together like an ‘adaptor’. To assess the superiority of imNA, T cell-mediated antitumor immunity was selected as an experimental model and four groups were established: (1) IgG isotype control (IgG control); (2) free αPD1 and αPDL1 (Free_αPD1 & αPDL1_); (3) a physical mixture of αFc-NP integrating αPD1 and αFc-NP integrating αPDL1 (NP_αPD1_ & NP_αPDL1_), and (4) αFc-NP simultaneously integrating αPD1 and αPDL1 (imNA_αPD1 & αPDL1_) (Fig. 3a). We first investigated the association of imNA_αPD1 & αPDL1_ with tumor cells and CD8^+^ T cells. To mimic the tumor microenvironment, B16-F10 melanoma cells and primary CD8^+^ T cells isolated from splenocytes were stimulated with interferon-gamma (IFN-γ) or anti-CD3/CD28 antibodies, and remarkable upregulation of PDL1 and PD1 was observed in B16-F10 cells and CD8^+^ T cells, respectively (Supplementary Fig. 7). Stimulated B16-F10 and CD8^+^ T cells were incubated with fluorescein isothiocyanate (FITC)-labeled imNA_αPD1 & αPDL1_, and the targeting ability was assessed via flow cytometry and microscopy. As shown in Supplementary Fig. 8, the amount of imNA_αPD1 & αPDL1_ associated with B16-F10 cells increased with the extension of incubation time, as measured by the elevated median fluorescence intensity (MFI). Importantly, we confirmed that most imNA_αPD1 & αPDL1_ was absorbed on the membrane but were not internalized into the cell via trypan blue quenching (Fig. 3b and Supplementary Fig. 8). Confocal laser scanning microscopy (CLSM) images also revealed that a substantial amount of imNA_αPD1 & αPDL1_ adsorbed onto the cell surface (the membrane of B16-F10 cells was labeled with the red dye PKH26) (Fig. 3c) rather than becoming internalized. Moreover, imNA_αPD1 & αPDL1_ also associated with CD8^+^ T cells in a time-dependent manner (Fig. 3d and Supplementary Fig. 9), and few formulations were internalized into CD8^+^ T cells (Fig. 3e). In contrast, αFc-NP integrating IgG control (αFc-NP_IgG_) exhibited weak interaction with both tumor cells and CD8^+^ T cells, indicating that the association of imNA_αPD1 & αPDL1_ with cells was dependent on specific antibody-antigen recognition (Fig. 3b, d, and Supplementary Figs. 8 and 9), and these results confirmed that the well-known co-inhibitory molecules PD1 and PDL1 can also serve as binding sites for imNA_αPD1 & αPDL1_. Additionally, FITC-labeled NP_αPDL1_ and NP_αPD1_ could only efficiently associate with B16-F10 cells and CD8^+^ T cells, but not both (Supplementary Fig. 10).

Having confirmed that imNA_αPD1 & αPDL1_ can associate with tumor cells and T cells simultaneously, we further investigated whether imNA_αPD1 & αPDL1_ could promote the effector-target cell conjugation. mCherry-expressing B16-F10 cells (B16-F10-mCherry) and carboxyfluorescein succinimidyl ester (CFSE)-labeled CD8^+^ T cells were coincubated in the presence of IgG control, Free_αPD1 & αPDL1_, NP_αPD1_ & NP_αPDL1_ or imNA_αPD1 & αPDL1_, and the conjugate formations were detected by confocal microscopy after washing suspended CD8^+^ T cells. As shown in Fig. 3f, imNA_αPD1 & αPDL1_ significantly increased the conjugation compared with other treatments, with approximately 60% of B16-F10 cells conjugated with one or more CD8^+^ T cells (Fig. 3g). As treatment with NP_αPD1_ & NP_αPDL1_ resulted in similar conjugate formations to IgG control and Free_αPD1 & αPDL1_, we confirmed that imNA_αPD1 & αPDL1_ could simultaneously tightly engage tumor cells and CD8^+^ T cells, and physically link them together. These results highlighted the critical distinction between imNA_αPD1 & αPDL1_ and the mixture of soluble mAbs or NP integrated with monospecific mAbs.

To investigate whether imNA_αPD1 & αPDL1_ could activate T cells in vitro, CD8^+^ T cells were coincubated with B16-F10 cells in a medium containing different forms of αPD1 and αPDL1, and cytokine production was examined at 24 h post-coincubation. imNA_αPD1 & αPDL1_-treated cells showed the highest level of interferon-gamma (IFN-γ), which is a predictor of cytotoxic T lymphocyte (CTL)-mediated response^31^, compared with cells treated with equivalent amounts of Free_αPD1 & αPDL1_ or NP_αPD1_ & NP_αPDL1_ (Fig. 3h). imNA_αPD1 & αPDL1_ also significantly increased the secretion of granzyme B and perforin^32^, the two main cytolytic granules released by CTLs (Fig. 3i, j). Next, a high content analysis (HCA) platform was utilized to determine the contribution of imNA_αPD1 & αPDL1_-facilitated cell interactions to CD8^+^ T cells cytotoxicity. Stimulated CD8^+^ T cells and B16-F10 cells were coincubated with the presence of different formulations and Annexin V-FITC, which is used to detect apoptotic cells. As revealed by HCA, Free_αPD1 & αPDL1_ and NP_αPD1_ & NP_αPDL1_ had minimal and slight effect on B16-F10 cell apoptosis (FITC-positive large cells), respectively, compared with IgG control. Intriguingly, imNA_αPD1 & αPDL1_ treatment drastically induced tumor cell upon extended incubation time, and few living tumor cells existed 48 h post-incubation (Fig. 3k, Supplementary Movie 1-4, and Supplementary Fig. 11). HCA images confirmed that the enhanced T cell-mediated cytotoxicity could be attributed to the imNA_αPD1 & αPDL1_-facilitated interaction between CD8^+^ T cells and tumor cells (Fig. 3l). Furthermore, we examined whether imNA_αPD1 & αPDL1_ could enhance the cytotoxicity of antigen-specific T cells by coculturing ovalbumin (OVA)-specific OT-1 CD8^+^ T cells and B16-F10-OVA cells in the presence of different formulations. As shown in Supplementary Fig. 12, imNA_αPD1 & αPDL1_-treated CD8^+^ T cells were more effective at killing B16-F10-OVA than those treated with a mixture of free antibodies or NP_αPD1_ & NP_αPDL1_. Collectively, these results confirmed that imNA_αPD1 & αPDL1_ can promote the interaction between CD8^+^ T cells and tumor cells, and achieve an enhanced antitumor activity in vitro over the combination of monospecific mAbs.

### imNA_αPD1 & αPDL1_ enhances the antitumor effect of T cells in vivo

**Fig. 4:**
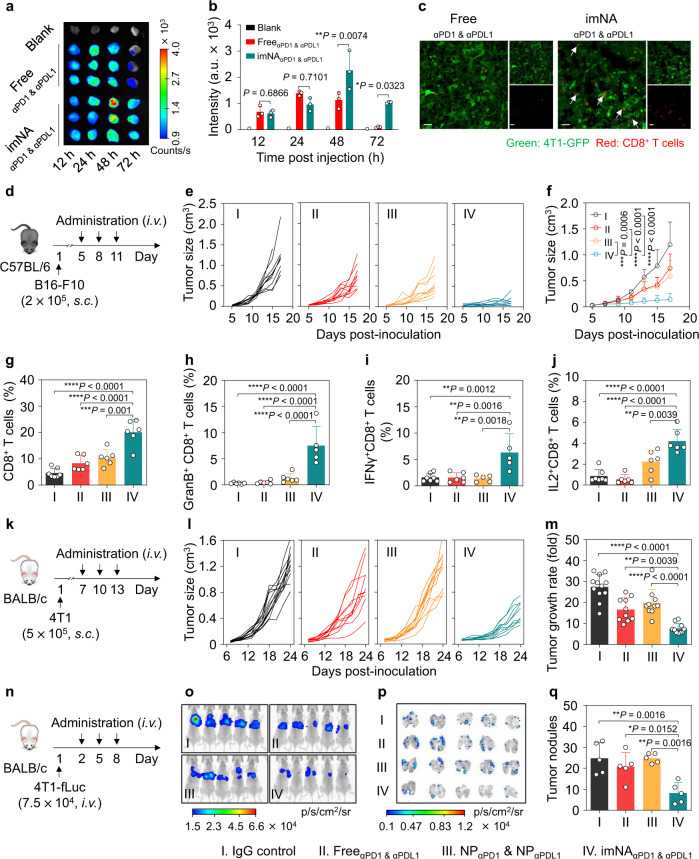
imNA_αPD1 & αPDL1_ exhibited superior antitumor efficacy in multiple murine tumor models. **a** IVIS imaging depicting the accumulation of imNA_αPD1 & αPDL1_ in 4T1 tumors. **b** Quantification of mAb accumulation in tumor tissues. Data are presented as means ± s.d. *n* = 3 biologically independent mice. Statistical significance was calculated via two-way ANOVA followed by the Tukey post-hoc test. *P < 0.05; **P < 0.01. **c** Engagement of 4T1-GFP tumor cells and CD8^+^ T cells in tumor tissues. Scale bars, 200 mm. **d** Experimental protocol for the B16-F10 melanoma model used in **e**–**g**, the equivalent injection dose of αPD1 and αPDL1 was 2.5 mg/kg. Individual (**e**), and average (**f**) tumor growth curves of B16-F10 tumors in different groups. Data are presented as means ± s.d. *n* = 10 biologically independent mice. **g** The abundance of CD8^+^ T cells in tumor tissues at the end of treatment. Data are presented as means ± s.d. *n* = 6-7 biologically independent mice (IgG control: *n* = 7; other groups: n = 6). Flow cytometry to evaluate the percentages of granzyme-secreting (**h**), IFN-γ-secreting (**i**), and IL-2-secreting CTLs (**j**) in melanoma tumors. Data are presented as means ± s.d. *n* = 5–7 biologically independent mice. For **h**, imNA_αPD1 & αPDL1_: *n* = 5, other groups: *n* = 6; for **i**, IgG control: *n* = 7, Free_αPD1 & αPDL1_: *n* = 6, other groups, *n* = 5; for **j**, IgG control: *n* = 7, other groups, *n* = 6. **k** Experimental protocol for the 4T1 breast cancer model used in **l**–**m**. **l** Individual tumor growth curves. **m** Tumor growth rates are shown in (**l**) at 24 days post-inoculation. Data are presented as means ± s.d. *n* = 10–12 biologically independent mice (IgG control: *n* = 12; other groups: *n* = 10). **n** Experimental protocol for the lung metastatic breast cancer model used in **o**–**q**. **o** In vivo bioluminescence imaging (BLI) of pulmonary metastases in mice at 16 days post-injection. **p** Ex vivo BLI of pulmonary metastases. **q** Tumor nodules on the lung tissues. Data are presented as mean ± s.d. *n* = 5 biologically independent mice. Statistical significance was calculated via one-way ANOVA with the Tukey post-hoc test. *P < 0.05; **P < 0.01; ***P < 0.001; ****P < 0.0001. Source data are provided as a Source Data file.

Encouraged by the capability of imNA_αPD1 & αPDL1_ to enhance CD8^+^ T cell-mediated cytotoxicity in vitro, we further assessed whether imNA_αPD1 & αPDL1_ could potentiate the antitumor effect of αPD1 and αPDL1 in vivo. Prior to the antitumor study, tumor enrichment of Free_αPD1 & αPDL1_ and imNA_αPD1 & αPDL1_ was first investigated. BALB/c mice bearing 4T1 breast tumors were intravenously administered with soluble or NP-immobilized Cy5-labeled αPD1 & αPDL1; tumor tissues were collected at predetermined time points, and the mAb signals were monitored using a fluorescence in vivo imaging system (IVIS). As shown in Fig. 4a, Free_αPD1 & αPDL1_ and imNA_αPD1 & αPDL1_ exhibited similar tumor accumulation at 12 h and 24 h post-injection. Notably, unlike the quick clearance of free mAbs, imNA_αPD1 & αPDL1_ continued to accumulate at the tumor sites after 24 h and were retained after more than 72 h. The imNA_αPD1 & αPDL1_ group showed ~100.3% and ~936.9% higher fluorescence intensity than the Free_αPD1 & αPDL1_ group at 48 h and 72 h, respectively (Fig. 4b). The intratumoral distribution of mAbs was also examined using immunofluorescence staining, and much stronger fluorescent signals were detected in the imNA_αPD1 & αPDL1_ group than the Free_αPD1 & αPDL1_ group at 48 h and 72 h, consistent with the results of IVIS imaging (Supplementary Fig. 13). Furthermore, mice bearing GFP-expressing 4T1 murine breast tumors were treated with Free_αPD1 & αPDL1_ or imNA_αPD1 & αPDL1_, and tumor tissues were harvested, sectioned, and stained with anti-CD8 antibody for confocal laser scanning microscopy (CLSM). As shown in Fig. 4c, CD8^+^ T cells (red) were mainly located in the space between tumor cells (green) in the Free_αPD1 & αPDL1_-treated tumors, while most CD8^+^ T cells were closely associated with tumor cells in the imNA_αPD1 & αPDL1_-treated group (as indicated by the white arrows), which was accompanied by enhanced cytotoxicity against tumor cells (measured by the destruction of cell integrity). These results confirmed that imNA_αPD1 & αPDL1_ can efficiently promote the engagement between T cells and tumor cells in vivo.

Next, an aggressive, hard-to-treat B16-F10 melanoma tumor model was established, and tumor-bearing mice were treated with IgG control, Free_αPD1 & αPDL1_, NP_αPD1_ & NP_αPDL1_ or imNA_αPD1 & αPDL1_ following a q3dx3 course (three times at intervals of three days) (Fig. 4d). As shown in Fig. 4e, f, Free_αPD1 & αPDL1,_ and NP_αPD1_ & NP_αPDL1_ only slightly suppressed tumor growth, with 37.8 ± 22.8% and 50.7 ± 25.50% inhibition rates *versus* the IgG control group at 18 days post-inoculation, respectively. In marked contrast, tumor growth in the imNA_αPD1 & αPDL1_ treated group was dramatically delayed and led to 4.3-fold and 3.2-fold smaller tumors compared with those receiving Free_αPD1 & αPDL1_ and NP_αPD1_ & NP_αPDL1_ treatments, respectively. It is noteworthy that the physical mixture of NP_αPD1_ and NP_αPDL1_ exhibited limited antitumor efficacy compared with imNA_αPD1 & αPDL1_, further corroborating the importance and necessity of immobilizing two mAbs onto a single NP. In addition, compared with the IgG control group, all the treatments improved median survival time of tumor-bearing mice, leading to a significantly longer time to endpoint in imNA_αPD1 & αPDL1_ group (Supplementary Fig. 14). Additionally, the selected mAb doses were well-tolerated in animal safety studies without noticeable weight loss during the treatment course (Supplementary Fig. 15).

To elucidate the mechanism by which imNA_αPD1 & αPDL1_ achieved improved antitumor activity, we sought to examine the frequency of the T cell subpopulation in tumor tissues. As shown in Fig. 4g and Supplementary Fig. 16, the frequency of CTLs (CD45^+^CD3^+^CD8^+^ T cells) in imNA_αPD1 & αPDL1_-treated tumors was 4.7-, 2.31-, and 1.81-fold higher than that of the IgG control, Free_αPD1 & αPDL1_, and NP_αPD1_ & NP_αPDL1_ groups, respectively. Meanwhile, imNA_αPD1 & αPDL1_ dramatically reduced the percentage of regulatory T cells (Tregs) (Supplementary Fig. 17), and the elevated CD8^+^ T cell/Treg ratio indicated that the imNA_αPD1 & αPDL1_ treatment could reverse the immunosuppressive microenvironment (Supplementary Fig. 18). More importantly, ex vivo phorbol 12-myristate 13-acetate/ionomycin (PMA) restimulation of T cells revealed that imNA_αPD1 & αPDL1_ could induce a substantial increase of Granzyme B-, IFN-γ (interferon-gamma)- and IL-2 (interleukin-2)-secreting CD8^+^ T cells relative to the other treatments, suggesting the enhanced antitumor functionality and proliferation of CTLs in imNA_αPD1 & αPDL1_-treated tumors (Supplementary Fig. 19 and Fig. 4h-j). We also found that T cells played a predominant role in the imNA_αPD1 & αPDL1_-mediated antitumor effect, while other PD1-expressing cells, including NK cells and DCs, played negligible roles (Supplementary Figs. 20 and 21). Furthermore, the PDL1-deficient B16-F10 cell line (PDL1-KO-B16-F10 cells) was constructed using CRISPR-Cas9 technology (Supplementary Fig. 22a, b). Notably, both Free_αPD1 & αPDL1_ and imNA_αPD1 & αPDL1_ exhibited marginal benefits in terms of tumor control in the subcutaneous PDL1-KO-B16-F10 model (Supplementary Fig. 22c), confirming the importance of PDL1 on tumor cells in the imNA-mediated anti-tumor response and the importance of imNA_αPD1 & αPDL1_-facilitated cell interaction in tumor therapy.

With the confirmation of the anti-melanoma effect, we further explored the general applicability of imNA_αPD1 & αPDL1_ using a murine 4T1 mammary tumor model, which emulates stage IV human breast cancer and is otherwise unresponsive to anti-PD1/PDL1 treatment^33^. Mice bearing orthotopic 4T1 tumors were treated as indicated above when the tumor volumes reached approximately 50 mm^3^ (Fig. 4k). At an equivalent injection dose, Free_αPD1 & αPDL1_, NP_αPD1_ & NP_αPDL1_ exhibited marginal benefits in terms of tumor control (Fig. 4l). Encouragingly, imNA_αPD1 & αPDL1_-treated mice showed an enhanced response rate (Fig. 4l) and a reduced tumor growth rate (Fig. 4m) compared to the control treatment; meanwhile, the changes in body weight during treatment were within acceptable limits (Supplementary Fig. 23). Furthermore, the potential of imNA_αPD1 & αPDL1_ to eliminate circulating tumor cells and inhibit tumor metastasis was evaluated in a pulmonary metastatic model. Mice were intravenously injected with 4T1 cells expressing firefly luciferase (4T1-fLuc) and then received a q3dx3 course from the second day (Fig. 4n). From the in vivo and ex vivo bioluminescence imaging results, in mice treated with PBS, Free_αPD1 & αPDL1_ and NP_αPD1_ & NP_αPDL1_, bioluminescence signals were evident on 16 days post-infusion, in contrast, mice treated with imNA_αPD1 & αPDL1_ exhibited the weakest bioluminescence signals (Fig. 4o, p). Direct observation of whole lungs and hematoxylin-eosin (H&E) staining verified the significant decrease in the number and size of metastatic nodules in the imNA_αPD1 & αPDL1_-treated group (Fig. 4q, and Supplementary Figs. 24, 25). The superior antimetastatic effects of imNA_αPD1 & αPDL1_ can be partly attributed to the imNA_αPD1 & αPDL1_-facilitated conjugation of CD8^+^ T cells and tumor cells in lung tissues and peripheral blood. Collectively, the enhanced antitumor efficacy of imNA_αPD1 & αPDL1_ in multiple tumor models indicated that imNA could extend the therapeutic potential of PD1/PDL1 blockade to a broader range of tumor types.

After confirming the remarkable antitumor effect of imNA_αPD1 & αPDL1_ in a variety of tumor models, we further investigated its biological safety. Male C57BL/6 mice (6–8 weeks old) were intravenously injected with Free_αPD1 & αPDL1_ or imNA_αPD1 & αPDL1_ (the injection dose of αPD1 and αPDL1 was 2.5 mg/kg) every three days, with three replicates. Six weeks following the final injection, mice were sacrificed to collect peripheral blood and the main organs. Multiple indices of liver function and renal function were examined. As shown in Supplementary Fig. 26, imNA_αPD1 & αPDL1_ treatment did not significantly impair liver or renal function. The main organs (including liver, spleen, lung, kidney colon, and intestine) were subjected to H&E staining and minor to-no lesions in these organs were observed via histological analyses (Supplementary Fig. 27).

### imNAs potentiate the antitumor effect of multiple immunomodulatory mAbs

**Fig. 5:**
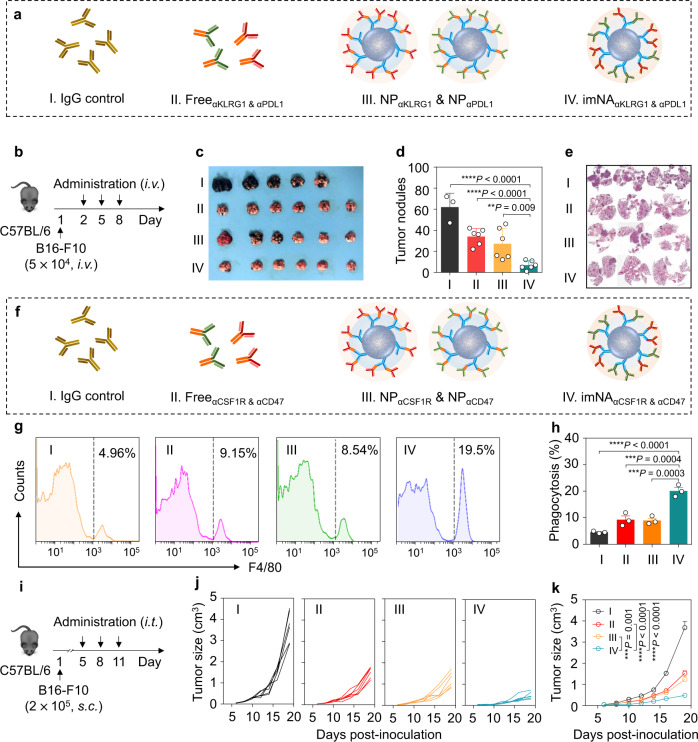
αFc-NP serves as a versatile antibody immobilization platform and imNAs enhance the anti-tumor effect of multiple immunomodulatory mAbs. **a** imNA was constructed to enhance the antitumor activity of NK cells, and the following four groups were established: (1) IgG control; (2) Free_αKLRG1 & αPDL1_; (3) NP_αKLRG1_ & NP_αPDL1_; and (4) imNA_αKLRG1 & αPDL1_. **b** Experimental protocol for the lung metastatic melanoma tumor model used in **c**–**e**: C57BL/6 mice were injected with 5.0 × 10^4^ B16-F10 melanoma cells via the tail vein, and treatment started on the second day. The equivalent dose of αKLRG1 and αPDL1 was 1.5 mg/kg. **c** Images of lung tissues at 20 days post tumor cell infusion. **d** Calculation of tumor nodules on the lung tissues. Data are presented as means ± s.d. *n* = 3 or 6 biologically independent mice, each pot represents one mouse. **e** Representative H&E images of lung tissues. **f** imNA was constructed to enhance the antitumor activity of macrophages, and the following four groups were established: (1) IgG control; (2) Free_αCSF1R & αCD47_; (3) NP_αCSF1R_ & NP_αCD47_; and (4) imNA_αCSF1R & αCD47_. Representative flow cytometric analysis images (**g**) and relative quantification (**h**) of the phagocytosis of cancer cells by bone marrow-derived macrophages (BMDMs). CFSE-labeled B16-F10 cells were incubated with BMDMs for 4 h in the presence of IgG control, Free_αCSF1R & αCD47_, NP_αCSF1R_ & NP_αCD47_ or imNA_αCSF1R & αCD47_, and then subjected to flow cytometric detection. Phagocytosis was quantified as the percentage of CFSE-positive BMDMs. Data are presented as means ± s.d. *n* = 3 biologically independent samples. **i** Experimental protocol for the B16-F10 melanoma model used in **j**, **k**, the equivalent dose of αCSF1R and αCD47 was 1.5 mg/kg, and the dose of IgG control was 3.0 mg/kg. Individual (**j**) and average (**k**) tumor growth curves of B16-F10 tumors in mice receiving different treatments. Data are presented as means ± s.d. *n* = 6 biologically independent mice. Statistical significance was calculated via one-way ANOVA with the Tukey post-hoc test. **P < 0.01; ***P < 0.001; ****P < 0.0001. Source data are provided as a Source Data file.

Having shown that imNA_αPD1 & αPDL1_ could significantly enhance the therapeutic efficacy of αPD1 and αPDL1 in vitro and in vivo, we next explored the possibility that imNAs could improve the antitumor efficiency of mAbs targeting immunomodulatory molecules expressed by tumor cells and other immune cells (for example, NK cells and macrophages). NK cells are potent cytotoxic lymphocytes of the innate immune system, and their activity is regulated by a repertoire surface receptor that recognizes their respective ligands on target cells^34,35^. Here, an NK cell-targeting imNA was constructed by gently mixing αFc-NP with anti-KLRG1 (killer-cell lectin-like receptor G1) antibody (αKLRG1) and anti-PDL1 antibody (αPDL1); the former could specifically blockade cadherin/KLRG1 interaction and enhance the cytolytic activity and proliferation of NK cells, and the latter can bind to tumor cells. To evaluate the superiority of imNA_aKLRG1 & αPD1_, a pulmonary metastatic model was established by i.v. injecting B16-F10 cells into C57BL/6 mice. Mice received treatment with IgG control, Free_αKLRG1 & αPDL1_, NP_αKLRG1_ & NP_αPDL1_ or imNA_aKLRG1 & αPD1_, with a q3dx3 course, and the administration doses of αKLRG1 and αPD1 were 1.5 mg/kg (Fig. 5a, b). Lungs from treated mice were harvested at 20 days post-injection. The observation of whole lungs (Fig. 5c) and calculation of metastatic nodules (Fig. 5d) showed that the formation of metastatic foci was effectively inhibited in the Free_αKLRG1 & αPD1_ (median, ~34) group and NP_αKLRG1_ & NP_αPDL1_ (median, ~27) group, compared with the control group (median, ~62). Encouragingly, very few metastatic foci (median, ~7) were observed in the lung tissues of imNA_αKLRG1 & αPDL1_-treated mice. H&E staining confirmed the significant decrease in the number and size of metastatic nodules in the imNA_αKLRG1 & αPDL1_ group (Fig. 5e). We further confirmed that the NK cells play a predominant role in the imNA_αKLRG1 & αPDL1_-mediated antitumor response using the NK cell depletion experiments (Supplementary Fig. 28).

Tumor-associated macrophages (TAMs) have attracted substantial attention in recent years, as they play a key role in tumor metastasis and therapeutic resistance^7^. TAMs are considerably plastic and can be either tumor-supportive (M2-like cells) or tumoricidal (M1-like cells). M2-like cells prevail in nearly all tumor types but have a limited ability to phagocytose tumor cells^36^. Additionally, cancer cells always express a ‘don’t eat me’ molecule, CD47, that binds to the signal regulatory protein α (SIRPα) receptor on macrophages and inhibits phagocytic activity^37^. We proposed that skewing the M2-like phenotype towards an M1-like lineage, thus blocking the ‘don’t eat me’ signal, and physically linking macrophages and tumor cells together could improve tumor cell engulfment. Thus, we immobilized αCSF1R (stimulating factor 1-receptor), which can inhibit CSF1/CSF1R signaling and repolarize the macrophage phenotype^38^, and αCD47 (anti-CD47 antibody) onto αFc-NP, to form a macrophage-targeting imNA (imNA_αCSF1R & αCD47_) (Fig. 5f). To evaluate the advantage of imNAαCSF1R & αCD47, we first cocultured CFSE-labeled B16-F10 cells and BMDMs (bone marrow-derived macrophages) in the presence of different therapeutics (Fig. 5g) and examined phagocytosis using flow cytometry. As shown in Fig. 5h, treatment with αCSF1R & αCD47 and NP_αCSF1R_ & NP_αCD47_ could improve phagocytosis as measured by the CFSE signal in the macrophages. Of note, the phagocytosis rate of the imNA_αCSF1R & αCD47_-treated group was substantially higher than those of the other groups. More importantly, imNA_αCSF1R & αCD47_-facilitated phagocytosis of tumor cells by macrophages can efficiently increase the cognate APC (antigen-presenting cell) function of macrophages and stimulate the clonal expansion of tumor-specific T cells (Supplementary Fig. 29).

Furthermore, we examined whether imNA_αCSF1R & αCD47_ could promote anti-tumor activity in vivo. Syngeneic mouse models of B16-F10 tumors implanted subcutaneously were established (Fig. 5i). Treatment with the imNA_αCSF1R & αCD47_ via an intratumoral injection at a predetermined dose showed a significant tumor-inhibition effect compared with Free_αCSF1R & αCD47_ and NP_αCSF1R_ & NP_αCD47_ (Fig. 5j, k). As expected, imNA_αCSF1R & αCD47_ administration also extended the survival time of tumor-bearing mice (Supplementary Fig. 30). Flow cytometry analysis and TAM-depleting experiments showed that the excellent tumor inhibitory effect was attributed to the improved phagocytosis of tumor cells by TAMs (Supplementary Figs. 31 and 32). Surprisingly, imNA_αCSF1R & αCD47_ could also reverse the immunosuppressive tumor microenvironment, as assessed by decreased M2 macrophages and increased M1 macrophages, T cells, and NK cells (Supplementary Fig. 33). Meanwhile, the absence of anemia after imNA_αCSF1R & αCD47_ administration validated the biological safety of our treatment regimens (Supplementary Fig. 34). Collectively, these results confirmed that αFc-NP could be a versatile and facile antibody immobilization platform; and multiple imNAs could be obtained by mixing αFc-NP with two types of well-chosen mAbs, and substantially improving the therapeutic efficacy of original mAbs.

## Discussion

Immunotherapy has attracted much more attention than traditional cancer treatment strategies (surgery, chemotherapy, radiation, and molecularly targeted therapy) in recent years, especially due to the remarkable success of immune checkpoint blockade in patients with a wide variety of malignancies^39^. As the predominant modality of cancer immunotherapy, immunomodulatory monoclonal antibodies (mAbs), including mAbs targeting co-inhibitory or co-stimulatory ligands/receptors of T cells and NK cells (e.g., PDL1, PD1, CTLA4, OX40, 4-1BB, NKG2D, TIGIT)^3,40^, mAbs re-educating or depleting TAMs (e.g., CSF1R, CCR2)^7^, and mAbs promoting the phagocytosis of macrophages and dendritic cells (CD47, SIRPα)^41,42^, may revolutionize the cancer treatment paradigm in the future. However, the clinical benefit of immunomodulatory mAbs is limited to a minority of patients due to innate and/or acquired resistance as well as their inevitable adverse effects^43^. Several approaches have been developed to improve the antitumor efficacies of immunomodulatory mAbs: (I) combining two types of mAbs with distinct mechanisms of action^44^; (II) combining mAbs with other treatment modalities^45^; and (III) increasing the tumor accumulation of mAbs via antibody modification or various delivery systems^45–47^. Undoubtedly, these approaches can improve the antitumor activities of mAbs to a certain extent; meanwhile, novel strategies that can broaden the clinical utility of immunomodulatory mAbs are still urgently needed.

Bispecific antibodies (bsAbs), which comprise two antigen-recognizing elements that are capable of simultaneously binding two distinct targets, are emerging as a promising format of cancer immunotherapy. Blinatumomab (CD3 × B lymphocyte antigen CD19) has been utilized to treat B-cell acute lymphocytic leukemia with impressive clinical results, and more than 50 bsAbs are currently being evaluated for the treatment of hematological and solid tumors^48^. The unique and highly advantageous feature of bsAbs over monospecific antibodies is the capability of triggering contact between effector cells and tumor cells and consequently enhancing cytotoxicity, which is unachievable for the mixture of monospecific antibodies. We speculated that integrating the features of mAbs (immunomodulating function of immune cells) and bsAbs (facilitating co-engagement of immune cells and tumor cells) into one system could dramatically boost immunotherapy, and we proposed that nanoparticle immobilizing two types of mAbs targeting effector cells and tumor cells could be such a system.

Emerging research has indicated that engineering multi mAbs onto nanoparticles may promote the interaction between effector cells and tumor cells, yet the underlying mechanism has not been fully elucidated^49^. More importantly, previously reported antibody immobilization approaches mainly rely on chemical reactions through primary amine groups, thiol groups, or sugar chains. However, these processes are difficult to control due to the high molecular weight and the multivalent properties of antibodies and nanoparticles, and they may also potentially hurt the valency of antibodies, limiting its clinical translation. Considering that all clinically approved mAbs for cancer immunotherapy are IgG isotypes comprising well-conserved Fc fragments, we proposed that nanoscale formulations decorated with anti-IgG (Fc specific) antibodies (αFc) could be a versatile antibody immobilization platform (αFc-NP), and it could theoretically immobilize any mAbs through Fc-specific recognition and interactions, which is utterly different from rough and complex chemical reactions. We successfully constructed αFc-NP via oriented conjugation of αFc onto nanoparticles and showed that αFc-NP can efficiently immobilize multiple therapeutic mAbs through gentle mixing. Disruption of antigen-binding capability (e.g., blockage of antigen-binding sites, destruction of antibody structure) is an intractable problem always encountered in antibody immobilization^29^. Encouragingly, without any chemical modification, mAbs can be immobilized by αFc-NP through noncovalent interactions. Additionally, the F(ab’)_2_ fragments of therapeutic mAbs face outward after immobilization, and their antigen-binding capacities are preserved to the greatest extent.

Be parallel to the facile construction process was the impressive antitumor efficacy of αFc-NP immobilizing two types of mAbs against effector cells and tumor cells (referred to as immunomodulating nano-adaptors, imNAs). We selected αPDL1 and αPD1 as model mAbs and validated that imNA_αPD1 & αPDL1_ could integrate the functions of mAbs and bsAbs. First, immobilized αPDL1 and αPD1 retained their intrinsic immunomodulatory properties and could reinforce the cytotoxicity of CD8^+^ T cells against tumor cells. Second, as imNA_αPD1 & αPDL1_ integrated αPDL1 against tumor cells and αPD1 against T cells, it could associate with both cells simultaneously and act as an ‘adaptor’ to tightly connect them. In addition, considering that multiple mAbs were integrated by single αFc-NP (known as multivalence), we predicted that imNA_αPD1 & αPDL1_ had a much stronger affinity for both cells compared with conventional bsAbs. Their amplified antitumor efficacy over the mixture of αPDL1 and αPD1 in vitro and in vivo indicated that imNA_αPD1 & αPDL1_-facilitated T cell/tumor cell interactions worked synergistically with the modulation of T-cell function by αPD1 & αPDL1. The general applicability of αFc-NP and therapeutic superiority of imNAs were further validated in natural killer cell- and macrophage-mediated antitumor immune responses using murine subcutaneous melanoma, orthotopic breast tumor, and lung metastasis models, encouraging the clinical translation of αFc-NP and imNAs. αFc-NP can potentially serve as a universal ‘adjuvant’ to various FDA (Food and Drug Administration)-approved mAbs and those being evaluated in clinical trials, facile mixing is expected to significantly improve the antitumor efficacies of mAbs. Additionally, although the present work focused on cancer immunotherapy, the versatile nanoplatform we reported here could be extended to the fields of targeted nanomedicine, disease diagnosis, and antibody engineering. For instance, bi-, tri-, or multi-specific antibodies can be constructed by mixing αFc-NP with certain mAbs instead of a sophisticated molecular design and genetic engineering^50^.

## Methods

### Materials

Amino-functionalized polystyrene nanoparticles (NP) (~120 nm in diameter) were purchased from Shanghai Macklin Biochemical Co., Ltd. Sodium periodate (NaIO_4_) was obtained from Shanghai Aladdin Bio-Chem Technology Co., Ltd. Sodium borohydride (NaBH_4_) was obtained from Energy Chemical (Shanghai, China). The goat anti-rat IgG (Fc specific) antibody (αFc) was obtained from Rockland Immunochemicals Inc. The *InVivo*Plus anti-mouse PD1 antibody (Clone: 29 F.1A12), anti-mouse PDL1 antibody (Clone: 10 F.9G2), anti-mouse KLRG1 antibody (Clone: 2F1), anti-mouse CD47 antibody (Clone: MIAP410), anti-mouse CSF1R antibody (Clone: AFS98) and rat IgG2a isotype control (anti-trinitrophenol, Clone: 2A3) were obtained from Bio X Cell. The fluorochrome-labeled antibodies for flow cytometry detection were purchased from BioLegend. The PKH26 Red Fluorescent Cell Linker Mini Kit, collagenase type I, hyaluronidase, and DNase I were obtained from Sigma-Aldrich. The CellTrace™ Blue Cell Proliferation Kit, CellTrace™ CFSE Cell Proliferation Kit, Alexa Fluor® 647 dye (AF647), and Alexa Fluor® 750 dye (AF750) was obtained from Thermo Fisher Scientific. Recombinant mouse PD1 and PDL1 proteins (rmPD1 and rmPDL1) were purchased from Sino Biological, Inc. Sulfo-Cyanine5 NHS ester and fluorescein isothiocyanate (FITC) were purchased from J&K Scientific. Cell and antibody labeling were performed according to the manufacturers’ protocols.

### Cell lines and animals

The B16-F10 murine melanoma cells and 4T1 murine mammary carcinoma cells were obtained from the American Type Culture Collection (ATCC). B16-F10 cells expressing membrane-bound chicken ovalbumin (B16-F10-OVA), B16-F10 cells expressing mCherry (B16-F10-mCherry), 4T1 cell expressing GFP, 4T1 cells expressing firefly luciferase (4T1-fLuc) were constructed by transfecting OVA-, mCherry-, GFP- or fLuc-encoding lentiviral vectors (Vectorbuilder) into B16-F10 or 4T1 cells. Cells were maintained in Dulbecco’s modified Eagle medium (B16-F10 cells) or RPMI-1640 medium (4T1 cells) supplemented with 10% fetal bovine serum (FBS, Gibco) and 1% penicillin/streptomycin (Invitrogen) in a humidified atmosphere containing 5% CO_2_ at 37 °C. Transduced cells were grown in medium containing the selection agent puromycin at a concentration of 0.25 μg/mL. Primary CD8^+^ T cells were isolated from the spleens of C57BL/6 or OT-1 transgenic mice using a CD8a (Ly2) microbeads isolation kit (Miltenyi Biotec.), and then cultured in RPMI-1640 medium supplemented with 10% FBS, 1% penicillin/streptomycin, 1% GlutaMAX (Life Technologies), 10 mM HEPES (Life Technologies), 1 mM sodium pyruvate (Life Technologies), 55 μM 2-mercaptoethanol (Life Technologies) and 10 ng/mL IL-2 (Peprotech). All cells were confirmed to be *Mycoplasma*-free using Hoechst DNA staining and agar culture methods.

Male C57BL/6 mice and female BALB/c mice were purchased from Hunan Silaikejingda Laboratory Animal Technology Co. Ltd. OT-I (C57BL/6-Tg (TcraTcrb) 1100 Mjb/J) TCR transgenic mice were a generous gift from Professor Tian-Meng Sun from Jilin University. All mice were maintained at the animal facility of South China University of Technology (SCUT) in a specific pathogen-free (SPF) environment with controlled temperature (~22 °C) and humidity (50 ± 15%) under 12 h light/dark cycle. Mice aged 6-8 weeks were used for experiments. All animal experiments were approved by the Animal Care and Use Committee at SCUT, and every effort was made to minimize suffering from experiments.

### αFc oxidation and αFc conjugation

The oxidation of carbohydrate residues on the Fc portion of αFc was performed by dissolving 1 mg/mL αFc in 50 mM acetate buffer (pH 4.2) containing sodium periodate (NaIO_4_, 10 mM) for 2 h at 4 °C, and oxidized αFc was recovered using Amicon® Ultra Centrifugal Filters (MWCO 100 kDa, Merck Millipore) (centrifugation at 9,000 × *g* for 5 min). The generation of aldehydes was detected by Purpald^®^ (4-amino-3-hydrazino-5-mercapto-1,2,4-triazole, Sigma). Then, oxidized αFc (0.1 mg/mL) was mixed with aminated NP at predefined mass ratios, and the reaction between primary amine and aldehyde groups was performed for 12 h at 4 °C with gentle stirring. Finally, sodium borohydride (NaBH_4_, Energy Chemical) was added to the mixtures and incubated for another 45 min to reduce the Schiff base intermediates and generate stable covalent linkages between NP and αFc. The fabricated formulations (αFc-NP) were collected by centrifugation, and the unbound αFc in supernatants was examined by enzyme-linked immunosorbent assay (ELISA) (goat IgG ELISA Kit, Alpha Diagnostic International) and ultra-performance liquid chromatography (UPLC). The binding efficacy (BE) of αFc was calculated using the following formula: BE = (A-B)/A, where A is the feeding amount of αFc and B is the αFc in the supernatant.

To measure the size distribution, NP and αFc-NP were diluted in a 5% glucose solution (1 mg/mL) and characterized using a Zetasizer Nano ZS instrument (Malvern, Inc.). For the morphology examination, NP and αFc-NP were drop cast onto silicon wafers and imaged using a field emission scanning electron microscope (Merlin Compact, Zeiss, Germany).

The method by which NP binds αFc was identified by reducing SDS-PAGE (sodium dodecyl sulfate-polyacrylamide gel electrophoresis). Briefly, free αFc or αFc-NP diluted in PBS (phosphate-buffered saline) was mixed with SDS-PAGE sample loading buffer (5×) (GeneCopoeia) and heated for 10 min at 99 °C. The reducing agent β-mercaptoethanol in sample loading buffer breaks the intrachain disulfide bonds of αFc and separates heavy chains and light chains. Then, the mixtures (20 µL, 500 μg/mL) were loaded onto polyacrylamide gels and run at 90 V for approximately 120 min, followed by staining with Coomassie Blue (Beijing Solarbio Science & Technology Co., Ltd). The bands of heavy chains (~50 kDa) and light chains (~25 kDa) were imaged with Typhoon Gel and Blot Imaging Systems (GE Healthcare) after destaining.

### Construction and characterization of αFc-NP/mAbs

αPD1 was mixed with αFc-NP at the αPD1/αFc ratio of 1:1 (w/w) and incubated for 12 h at 4 °C, and the diameter of αFc-NP_αPD1_ was examined using Zetasizer Nano ZS instrument (Malvern, Inc.). To confirm that αPD1 was immobilized onto αFc-NP via Fc-specific recognition, the IgG isotype control or αFc was conjugated to AF750-labeled NP, followed by incubation with AF647-labeled αPD1. The mixtures (IgG-NP_αPD1_ or αFc-NP_αPD1_) were dropped on the coverslip and precipitated for 10 min, and images were acquired using a SRiS STORM Super-resolution System (NanoBioImaging, Ltd.). SRiS is equipped with 647 nm and 750 nm super-resolution compatible laser excitation, which enables industry-leading two-color simultaneous STORM acquisition.

To confirm that αFc-NP could integrate two types of mAbs simultaneously, PerCP-Cy5.5-conjugated αPDL1 and FITC-conjugated αPD1 (BioLegend) were incubated alone or in combination with αFc-NP for 12 h and then subjected to high-sensitivity nanoflow cytometry (HSFCM, NANOFCM CO., LTD). The HSFCM instrument was equipped with three single-photon-counting avalanche photodiode (APD) detectors for the simultaneous detection of side scattering (SSC) and two-color fluorescence. The acquired data were analyzed by FlowJo v10 (Tree Star).

To detect stability, αFc-NP_αPD1_ was incubated with 5% glucose for 48 h and the diameter variation was examined using a Zetasizer Nano ZS instrument (Malvern, Inc.). To investigate the stability of αFc-NP_αPD1_ in the presence of IgG from the same or different host species, αFc-NP integrating PerCP-Cy5.5-labeled PDL1 (αFc-NP_PP-Cy5.5-αPDL1_) was incubated with the rat IgG control and mouse IgG control, and the concentrations of PerCP-Cy5.5-αPDL1, rat IgG control, and mouse IgG were 10 μg/mL. The percentage of PerCP-Cy5.5^+^ αFc-NP_PP-Cy5.5-αPD1_ after 12 h and 24 h incubation was examined by nanoflow cytometry (HSFCM, NANOFCM CO., LTD).

### αPD1 and αPDL1 binding efficiency

αPD1 or αPDL1 were mixed with αFc-NP (the mass ratio of αPD1 and αPDL1 to αFc was 1:1) and incubated at 4 °C for a predetermined time (1, 2, 4, 8, 12, 24 h). The solution was centrifuged at 20,000 × *g* for 60 min, and the unbound αPD1 and αPDL1 in the supernatant were examined by ELISA. Briefly, 96-well plates (Corning) were coated with 100 μL (5 μg/mL) rmPD1 or rmPDL1 for 2 h at 37 °C, followed by blocking with 2% BSA (bovine serum albumin, Sigma) in PBST (1 × PBS containing 0.1% Tween® 20) for 1 h at room temperature (RT). Samples were then incubated with free αPD1 or αPDL1 (a series of concentration gradients) or the collected supernatant for 1 h at RT. After washing, 100 μL (1 μg/mL) HRP-conjugated goat anti-rat IgG antibody (Sino Biological, Inc.) was added as the detection antibody and incubated for 1 h at RT, followed by the addition of 100 µL of 3,3′,5,5′-tetramethylbenzidine (TMB, Abcam). After another 10 min incubation, a 450 nm stop solution for the TMB substrate was added, and the absorbance intensity in each well was detected using an 800 TS microplate reader (Biotek).

To examine whether αFc-NP could bind two types of mAbs in a controlled manner, αPD1 and αPDL1 (the mass ratios of αPD1 to αPD1 ranged from 1/3 to 3) were incubated with αFc-NP (the mass ratio of αPD1 & αPDL1 to αFc was 1:1) for 12 h at 4 °C. The solution was centrifuged at 20,000 × *g* for 60 min, and the unbound αPD1 and αPDL1 in the supernatant were examined by ELISA as indicated above.

### Antigen-binding capability

ELISA plates (Corning) were coated with 100 μL (5 μg/mL) of rmPD1 or rmPDL1 for 2 h at 37 °C, followed by blocking with 2% BSA in PBST for 1 h at RT. A series of concentrations of free mAbs (αPD1 or αPDL1) or NP-immobilized mAbs (αFc-NP_αPD1_ or αFc-NP_αPDL1_) were added and incubated for 1 h at RT. The attached antibodies were examined as indicated above. The dissociation constant K_D_ was obtained by plotting normalized absorbance values versus the concentrations of αPD1 or αPDL1 using PRISM software (GraphPad).

### Stimulation of tumor cells and CD8^+^ T cells in vitro

B16-F10 or B16-F10-mCherry cells were stimulated with IFN-γ (20 ng/ml, Peprotech) for 24 h. Murine spleens were carefully removed and washed three times with sterile PBS on ice. The spleens were gently fragmented between glass microscope slides and the splenocyte suspension was filtered through a 40-μm nylon mesh. Red blood cells were removed using ACK (Ammonium-Chloride-Potassium) Lysing Buffer (BioLegend), the splenocytes were then resuspended in magnetic-activated cell sorting (MACS) buffer and CD8^+^ T cells were isolated via a CD8a (Ly2) microbeadsIsolation Kit (Miltenyi Biotec.). For T cell stimulation, isolated CD8^+^ T cells were incubated with plate-bound anti-CD3 antibodies (5 μg/mL, BioLegend) and soluble anti-CD28 antibodies (5 μg/mL, BioLegend) for 48 h. PD1 or PDL1 expression after stimulation was assessed using a BD FACSCelesta™ flow cytometer (BD Biosciences).

### Association between cells and imNA_αPD1 & αPDL1_

Briefly, 1.0 × 10^5^ stimulated B16-F10 cells or CD8^+^ T cells were seeded into 24-well plates and incubated overnight, followed by the addition of αFc-NP immobilizing IgG control (αFc-NP_IgG_) or αFc-NP immobilizing αPD1 and αPDL1 (imNA_αPD1 & αPDL1_). NPs were labeled with FITC, and the concentration of αPD1 and αPDL1 was 10 μg/mL. After timed intervals, cells were washed three times with ice-cold PBS, and the mean fluorescence intensity (MFI) of FITC from B16-F10 cells or CD8^+^ T cells was detected on a BD FACSCelesta™ flow cytometer (BD Biosciences). For the distinction between internalized and surface‐bound αFc-NP_IgG_ or imNA_αPD1 & αPDL1_, trypan blue (0.4%, Thermo Fisher Scientific), which has been demonstrated to quench the fluorescence of FITC-labeled compounds when in close contact with them, was used to quench surface‐bound fluorescence and added to the cell suspension before FACS acquisition. The surface-bound fluorescence (SBF) was calculated using the following equation: SBF = A−B, where A and B represent the MFI without or with trypan blue quenching, respectively.

To directly visualize the association between cells and imNA_αPD1 & αPDL1_, B16-F10 cells or CD8^+^ T cells were labeled with red fluorescent dye using the PKH26 Red Fluorescent Cell Linker Kit (Sigma) according to the manufacturer’s protocol and then incubated with FITC-labeled imNA_αPD1 & αPDL1_ for 12 h, followed by the observation using a laser scanning confocal microscope (Zeiss).

### imNA_αPD1 & αPDL1_ facilitates the interaction between tumor cells and CD8^+^ T cells

B16-F10-mCherry cells and isolated CD8^+^ T cells were stimulated as indicated above. B16-F10-mCherry cells were seeded into Nunc™ glass-bottom dishes (Thermo Fisher Scientific) at a density of 5.0 × 10^3^ cells per dish, and 5.0 × 10^4^ CFSE-labeled CD8^+^ T cells were added 4 h later. Cocultured cells were treated with IgG control (20 μg/mL), Free_αPD1 & αPDL1_, NP_αPD1_ & NP_αPDL1_ or imNA_αPD1 & αPDL1_; the concentration of αPD1 and αPDL1 was 10 μg/mL. After an additional 8 h incubation, suspended CD8^+^ T cells were removed, and T cell-tumor cell conjugations were visualized under a Zeiss LSM880 laser scanning confocal microscope. The conjugation rates, measured as the percentage of B16-F10 cells conjugating one or more CD8^+^ T cells, were determined manually by observing red/green contact in multiple nonoverlapping images using ImageJ v1.47 (National Institutes of Health, USA).

### Detection of IFN-γ, granzyme B, and perforin via ELISA

A total of 5.0 × 10^3^ B16-F10 cells and 5.0 × 10^4^ stimulated CD8^+^ T cells were seeded into 96-well plates (Corning) and incubated overnight. Cocultured cells were treated with IgG control (20 μg/mL), Free_αPD1 & αPDL1_, NP_αPD1_ & NP_αPDL1_ or imNA_αPD1 & αPDL1_, the concentration of αPD1 and αPDL1 was 10 μg/mL. At 24 h post-treatment, the supernatant was collected, and the modulators of T cell-mediated cytotoxicity in the supernatant were quantified via Mouse IFN-γ ELISA Kit (Dakewe Biotech), Mouse Granzyme B ELISA Kit (Abcam), and Mouse Perforin 1 ELISA Kit (Abbexa) according to the manufacturer’s protocol.

### In vitro apoptosis assay

To examine the cytotoxicity of nonantigen-specific T cells, B16-F10-mCherry cells were seeded into CellCarrierUltra ULA 96-well microplates (PerkinElmer) at a density of 5.0 × 10^3^ cells per well and allowed to adhere for 12 h, and 5.0 × 10^4^ stimulated CD8^+^ T cells labeled with CellTrace Blue were then added. Cocultured cells were treated with IgG control (20 μg/mL), Free_αPD1 & αPDL1_, NP_αPD1_ & NP_αPDL1_ or imNA_αPD1 & αPDL1_; the concentration of αPD1 and αPDL1 was 10 μg/mL. FITC conjugated recombinant Annexin V (Annexin V-FITC, Thermo Fisher Scientific) was added to the medium (final concentration was 1 μg/mL) to detect apoptotic cells. The plates were incubated at 37 °C and 5% CO_2,_ and images of cocultured cells were continuously acquired every 45 min using the Operetta CLS™ High-Content Analysis System (PerkinElmer) for 48 h. The viability of B16-F10 cells was evaluated using Harmony^®^ high-content analysis software based on cellular phenotypes and fluorescence distribution parameters.

To examine the cytotoxicity of antigen-specific T cells, CD8^+^ T cells isolated from OT-1 transgenic mice were cultured and stimulated with anti-CD3/28 antibodies (5 μg/mL) for 24 h. CD8^+^ T cells were then cocultured with Hoechst 33342 (H33342)-labeled B16-F10-OVA cells at ratios of 5:1 or 10:1 in 100 µL media in the presence of the IgG control antibody (20 μg/mL), Free_αPD1 & αPDL1_, NP_αPD1_ & NP_αPDL1_ or imNA_αPD1 & αPDL1_ for 48 h, and the concentration of αPD1 and αPDL1 was 10 μg/mL. After co-incubation, the amount of H33342 in the supernatant and adherent cells was examined using an Infinite® 200 PRO microplate plate reader, and cell viability was calculated using the following formula: cell viability = (A-B)/A, where A is the total amount of H33342, and B is the H33342 in the supernatant.

### Tumor accumulation of imNA_αPD1 & αPDL1_ and cell interaction in vivo

To investigate the tumor accumulation of imNA_αPD1 & αPDL1_, orthotopic breast tumor models were established by injecting 5.0 × 10^5^ 4T1 cells into the mammary fat pad of female BALB/c mice (6-8 weeks old). Mice were randomly divided into three groups when the tumor volume reached approximately 300 mm^3^, followed by intravenous administration of PBS, Free_αPD1 & αPDL1_ or imNA_αPD1 & αPDL1_; αPD1 and αPDL1 were labeled with Cy5 dye (Thermo Fisher Scientific) and the injection dose of Cy5-labeled αPD1 & αPDL1 was 5.0 mg per kg mouse body weight. Mice were sacrificed at predetermined time intervals, and tumor tissues were harvested for fluorescence imaging using the In-Vivo Xtreme II imaging system (Bruker). The acquired images were analyzed using Molecular Imaging Software (Bruker). Next, tumor tissues were fixed in 4% paraformaldehyde (Sigma) overnight at 4 °C, immersed in 30% sucrose solution overnight, and then sectioned into 10 μm pieces and stained with DAPI (4′,6-diamidino-2-phenylindole) for confocal microscopy observation (Zeiss).

To validated whether imNA_αPD1 & αPDL1_ could enhance the interaction between CD8^+^ T cells and tumor cells, mice bearing 4T1-GFP tumors were treated with Free_αPD1 & αPDL1_ or imNA_αPD1 & αPDL1_ (5 mg/kg, every three days for three times), 24 h post-final injection, tumor tissues were harvested and stained with anti-CD8a antibody and Alexa Fluor® 568 labeled goat anti-rabbit IgG H&L antibody, followed by confocal observation.

### Anti-tumor study

For the subcutaneous melanoma model, 2.0 × 10^5^ B16-F10 cells in 100 μL of PBS were subcutaneously inoculated into the right flank of the 6-8-week-old female C57BL/6 mice. For the orthotopic breast tumor model, 5.0 × 10^5^ 4T1 cells were injected into the mammary fat pads of female BALB/c mice. When the tumor volumes were approximately 50 mm^3^, mice were randomly divided into four groups (6 or 10 mice per group) and intravenously or intratumorally administered the corresponding formulations on a schedule of an injection every 3 days for a total of three injections (q3dx3). For imNA_αPD1 & αPDL1_, the doses of αPD1 and αPDL1 were 2.5 mg/kg, while for imNA_KLRG1 & αPDL1_ and imNA_αCSF1R & αCD47_, the doses of αKLRG1, αPDL1, αCSFR1 and αCD47 were 1.5 mg/kg. Tumor volumes were monitored by measuring the perpendicular diameter with a caliper, and the estimated volume was calculated based on the following equation: *V* = *L* × *W*^2 ^× 1/2 (*V*, volume; *L*, length; *W*, width of tumor). Body weights were monitored every three days. The survival rates were expressed using Kaplan–Meier survival analysis.

For the lung metastasis model, 7.5 × 10^4^ 4T1-fLuc cells or 5.0 × 10^4^ B16-F10 cells were administered via the tail vein into female BALB/c or C57BL/6 mice, and treatments were initiated the following day as indicated above. For the bioluminescence observation, mice were intraperitoneally administered 3 mg of the D-luciferin (Dalian Meilun) in 200 µL of PBS, followed by the observation via In-Vivo Xtreme II imaging system (Bruker). On day 16, BALB/c mice were sacrificed and the lungs were separated for bioluminescence imaging after soaking in the D-luciferin solution. On day 20, C57BL/6 mice were sacrificed, and the lungs were separated. The metastatic foci in lung tissues were counted and recorded. Then, lung tissues were fixed in 4% paraformaldehyde (Sigma) and embedded in paraffin; the paraffin-embedded lungs were cut and stained with hematoxylin-eosin for immunohistochemical analysis.

### Flow cytometry analysis

Tumor tissues were harvested, minced, and incubated with RPMI-1640 medium containing 10% FBS (v/v), collagenase type I (1 mg/mL), hyaluronidase (100 μg/mL) and DNase I (100 μg/mL) at 37 °C for 25 min with persistent agitation. Digested cells were passed through a 40-μm nylon mesh and collected by centrifugation at 500 g for 10 min, followed by red blood cell (RBC) lysis. One hundred microliters of cell suspension (2.0 × 10^7^ cells/mL) were used for flow cytometry detection. For the analysis of T cell subpopulations, cells were stained with the following antibody cocktails: Alexa Fluor® 700-conjugated antibody to CD45 (Clone: 30-F11, dilution: 1:200), FITC-conjugated antibody to CD3 (Clone: 17A2, dilution: 1:400), BV650-conjugated antibody to CD8 (Clone: 53-6.7, dilution: 1:200), PE/Dazzle 594-conjugated antibody to CD4 (Clone: GK1.5, dilution: 1:200), and PE-conjugated antibody to CD25 (Clone: 3C7, dilution: 1:100). BV, brilliant violet; FITC, fluorescein isothiocyanate; PE, phycoerythrin.

For intracellular cytokine staining, 2.0 × 10^6^ tumor-infiltrating lymphocytes were seeded into 6-well plates in RPMI-1640 medium containing 10% FBS and supplemented with eBioscience™ Cell Stimulation Cocktail (plus protein transport inhibitors). After stimulation, cells were stained for surface markers; fixed and permeabilized with Intracellular Staining Permeabilization Wash Buffer (BioLegend); followed by staining with PerCP-Cy5.5-conjugated antibody to IL2 (Clone: JES6-5H4, dilution: 1:200), PE-conjugated antibody to IFN-γ (Clone: XMG1.2, dilution: 1:200) and Alexa Fluor® 647-conjugated antibody to Granzyme B (Clone: GB11, dilution: 1:200). All samples were acquired on a BD LSRFortessa™ flow cytometer, and Data were analyzed using FlowJo v10 (Tree Star).

### Statistics and reproducibility

All experiments have been reproduced at least two times, and all attempts at replication were successful with self-consistent results. All results are presented as means ± standard deviation (s.d.), and differences with *P* < 0.05 were considered significant. One-way and two-way analyses of variance (ANOVA) with a Tukey post-hoc test was used for multiple comparisons, and Student’s *t*-test was used for two-group comparisons. Survival curves were analyzed using the log-rank (Mantel–Cox) test. Significance levels were defined as ns (not significant, *P* > 0.05), *P < 0.05, **P < 0.01, ***P < 0.001 and ****P < 0.0001, *P* values were calculated by GraphPad Prism 7 (GraphPad Software, Inc.) and marked on the figures.

### Reporting summary

Further information on research design is available in the Nature Research Reporting Summary linked to this article.

## Supplementary information

Supplementary Information

Description of Additional Supplementary Files

Supplementary Movie 1

Supplementary Movie 2

Supplementary Movie 3

Supplementary Movie 4

Reporting summary

## Data Availability

All data are available within the Article, Supplementary Information, or available from the authors upon request. Source data are provided with this paper.

## Source data

Source Data
