# Supplementary material for: Immunomodulating nano-adaptors potentiate antibody-based cancer immunotherapy
Source: Nat Commun. 2021 Mar 1;12:1359. doi: 10.1038/s41467-021-21497-6 (PMC7921676; doi:10.1038/s41467-021-21497-6)
Supplement: Supplementary file 1 — Supplementary Information [file 41467_2021_21497_MOESM1_ESM.pdf]

Supplementary Information

for

Immunomodulating nano-adaptors potentiate antibody-based cancer immunotherapy

Cheng-Tao Jiang, Kai-Ge Chen, An Liu, Hua Huang, Ya-Nan Fan, Dong-Kun Zhao, Qian-Ni Ye, Hou-Bing Zhang, Cong-Fei Xu, Song Shen*, Meng-Hua Xiong, Jin-Zhi Du, Xian-Zhu Yang, Jun Wang*

Correspondence should be addressed to Prof. S. Shen (shensong@scut.edu.cn) and Prof. J. Wang (mcjwang@scut.edu.cn)

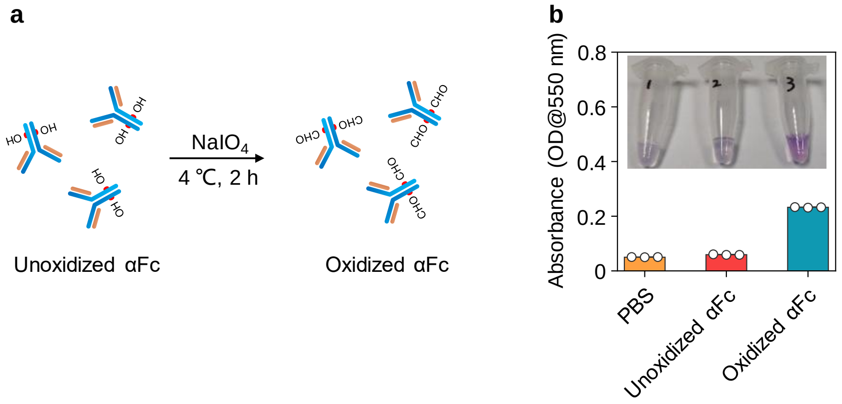

Supplementary Figure 1 | Oxidation of anti-IgG (Fc specific) antibody (αFc). a The oxidation of carbohydrate residues on the Fc portion of αFc was performed by dissolving 1 mg/mL αFc in 50 mM acetate buffer (pH 4.2) containing sodium periodate ($NaIO_4$, 10 mM) for 2 h at 4 °C. **b** The generation of aldehydes was detected by Purpald® (4-amino-3-hydrazino-5-mercapto-1,2,4-triazole). The Purpald-aldehyde adduct is deep purple in color and has an absorption maximum at 550 nm. Data were presented as mean ± s.d. n = 3 biologically independent samples.

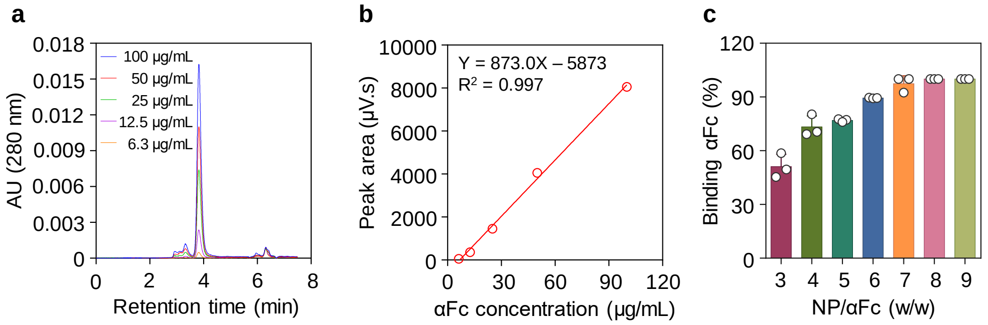

Supplementary Figure 2 | Binding efficacy of αFc examined by UPLC. The unbound αFc in supernatant was analyzed by ultra-performance liquid chromatography (UPLC, Waters) equipped with Protein BEH SEC Column (250 Å). **a** Representative chromatograms of αFc of different concentrations. The detection wavelength was 280 nm. **b** Calibration of peak area under the peak across retention time from 3.55 min to 4.64 min in UPLC analysis against concentration of αFc standard, the peak area was increased in proportion to the concentration of αFc, which revealed a significant correlation ($R^2 = 0.997$). **c** Binding efficacy of αFc at different NP/αFc mass ratio. Data were presented as the mean ± s.d. n = 3 biologically independent samples.

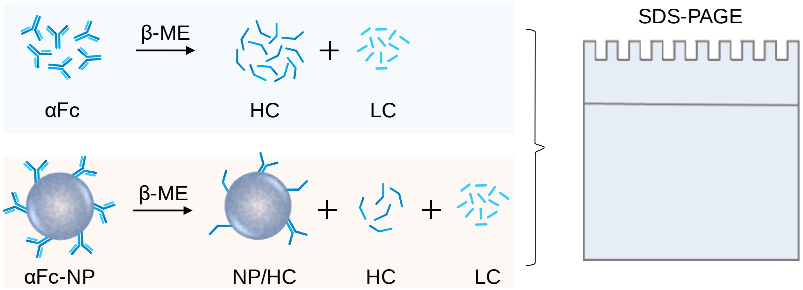

Supplementary Figure 3 | Reducing SDS-PAGE. β-mercaptoethanol (β-ME, a reducing agent) treatment could separate the heavy chains (HC) and light chains (LC) of αFc by breaking the inter-chain disulfide bonds. If αFc was chemically conjugated to NP *via* carbohydrate residue, partial HC was still attached to the NP after β-ME treatment and could not enter the gel matrix during SDS-PAGE process.

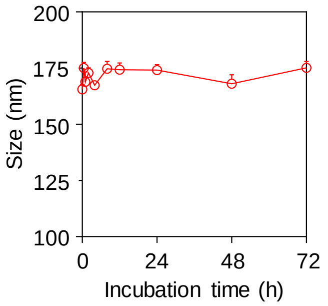

Supplementary Figure 4 | Stability of αFc-NP$_{αPD1}$. αFc-NP$_{αPD1}$ was incubated with 5% glucose solution for different periods, and the size variation was examined by the Zetasizer Nano ZS Instrument. Data were presented as the mean ± s.d. n = 3 biologically independent samples.

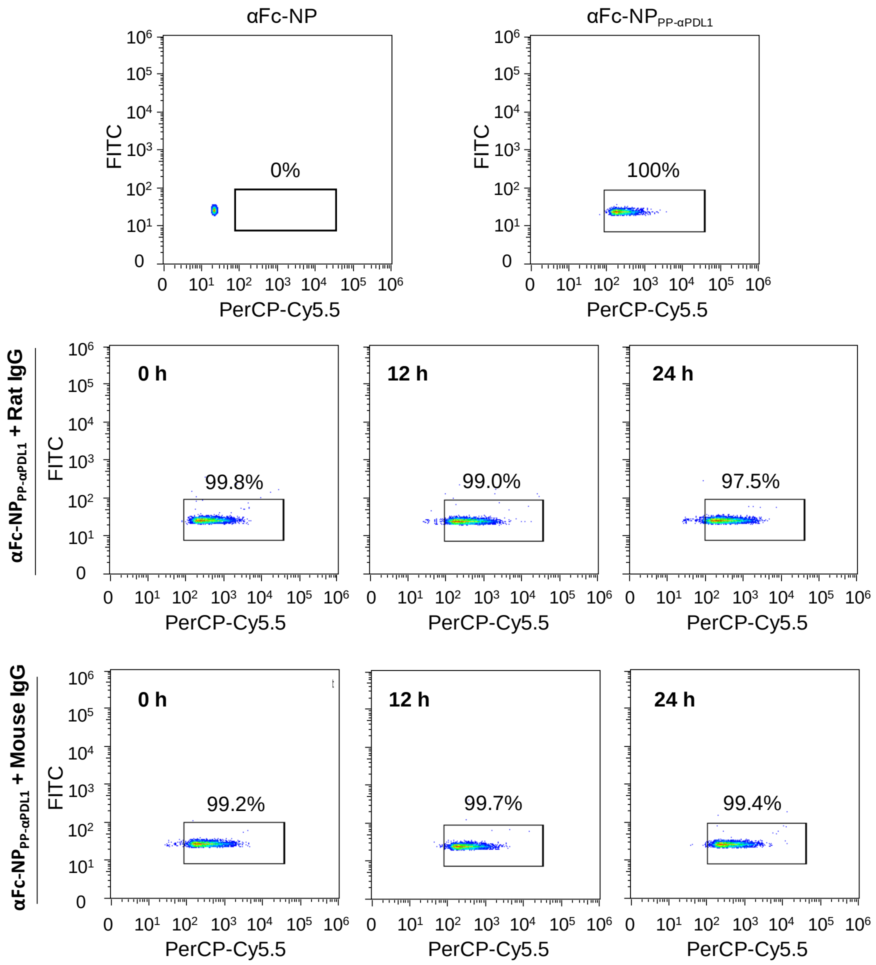

Supplementary Figure 5 | Stability of αFc-NP$_{\alpha PD1}$ with the presence of IgG from other species. αFc-NP integrating PerCP-Cy5.5-labeled αPDL1(αFc-NP$_{PP\text{-}PDL1}$) was incubated with rat IgG control and mouse IgG control for 12 h and 24 h and then subjected to Nano Flow Cytometry. The percentages of PerCP-Cy5.5 negative formulations indicated the replacement of PerCP-Cy5.5-αPDL1 by mAbs from other species.

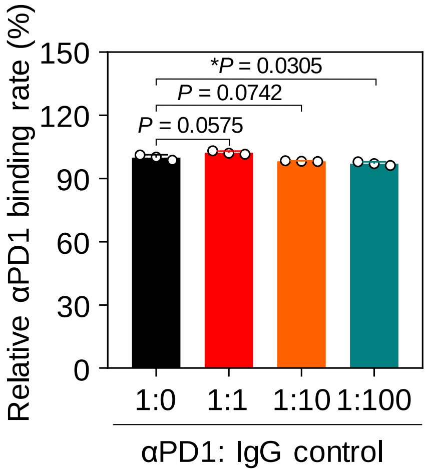

Supplementary Figure 6 | αFc-NP$_{\alpha PD1}$ was incubated with different concentrations of rat IgG isotype control antibody (the ratios of αPD1: IgG control range from 1:1 to 1:100) for 12 h, the binding rate of αPD1 post-incubation was examined by ELISA. Data were presented as the mean ± s.d. Statistical significance was calculated *via* one-way ANOVA with a Tukey post-hoc test. *$P <$ 0.05. Data were presented as mean ± s.d. n = 3 biologically independent samples.

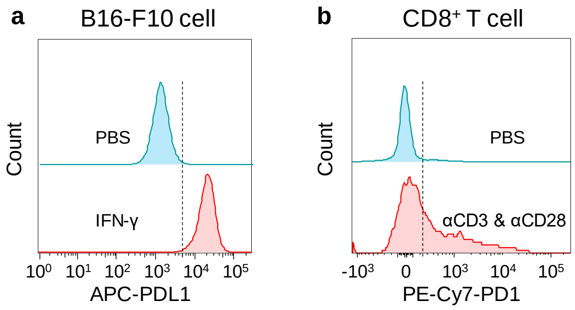

Supplementary Figure 7 | *In vitro* stimulation of B16-F10 melanoma cell and CD8$^+$ T cell. B16-F10 melanoma cells and primary CD8$^+$ T cells were stimulated with IFN-γ (20 ng/mL) and αCD3 & αCD28 (5 μg/mL) for 24 h, respectively. The expressions of PDL1 (**a**) and PD1 (**b**) were analyzed by flow cytometry.

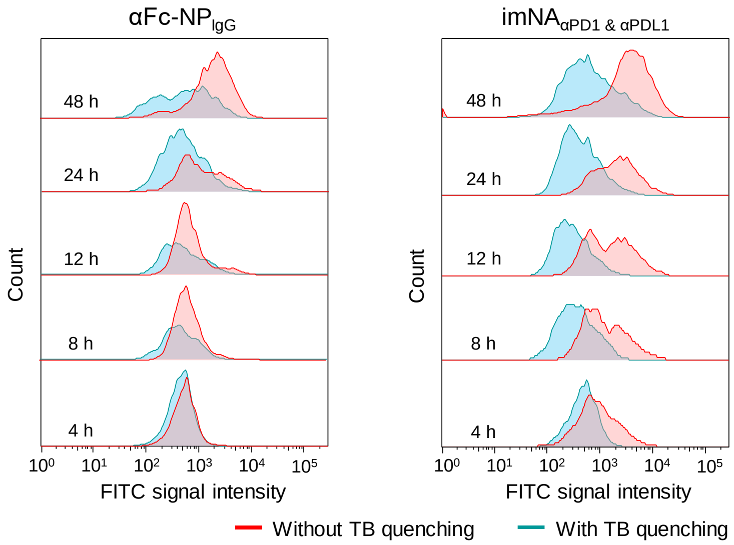

Supplementary Figure 8 | Association of imNA$_{\mathbf{aPD1\ \&\ aPDL1}}$ with B16-F10 cells. Stimulated B16-F10 cells were incubated with αFc-NP$_{IgG}$ or imNA$_{αPD1\ \&\ αPDL1}$ for 4, 8, 12, 24, 48 h; NP was labeled with FITC. At the predetermined time point, B16-F10 cells were collected, and the FITC signal was analyzed by flow cytometry without (red lines) and with (blue lines) trypan blue exclusion.

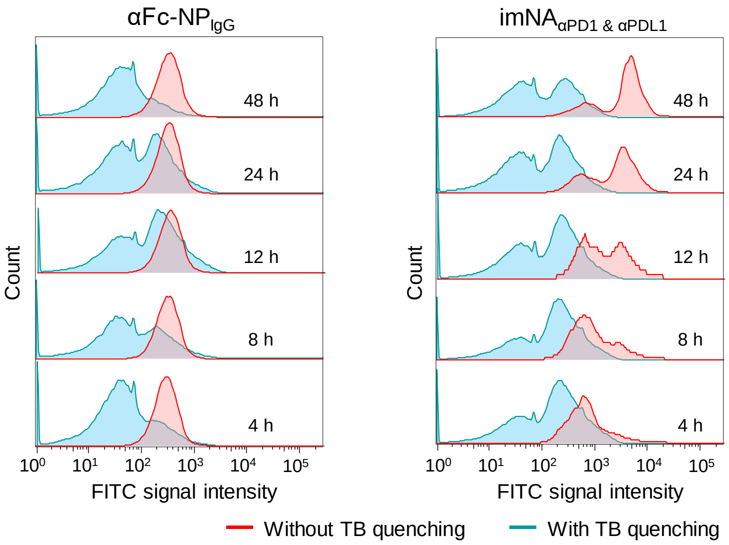

Supplementary Figure 9 | Association of imNA$_{αPD1 \& αPDL1}$ with CD8$^+$ T cells. Stimulated CD8$^+$ T cells were incubated with αFc-NP$_{IgG}$ or imNA$_{αPD1 \& αPDL1}$ for 4, 8, 12, 24, 48 h; NP was labeled with FITC. At the predetermined time point, CD8$^+$ T cells were collected, and the FITC signal was analyzed by flow cytometry without (red line) and with (blue line) trypan blue exclusion.

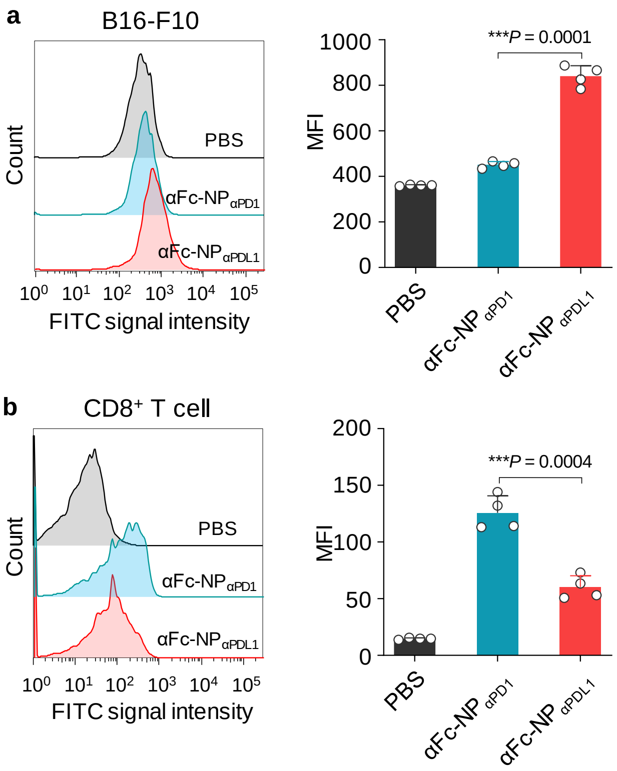

Supplementary Figure 10 | Association of $NP_{\alpha PD1}$ or $NP_{\alpha PDL1}$ with B16-F10 cells or $CD8^+$ T cells. $NP_{\alpha PD1}$ or $NP_{\alpha PDL1}$ was incubated with cocultured B16-F10 cells and $CD8^+$ T cells for 8 h, and analyzed by flow cytometry. The concentration of αPD1 or αPDL1 was 25 μg/mL. **a** Histogram, and mean fluorescence intensity (MFI) of FITC signal in B16-F10 cells. **b** Histogram, and MFI of FITC signal in $CD8^+$ T cells. Data were presented as mean ± s.d. n = 4 biologically independent samples. Statistical significance was calculated *via* an unpaired two-tailed Student's *t* test. ***P< 0.001.

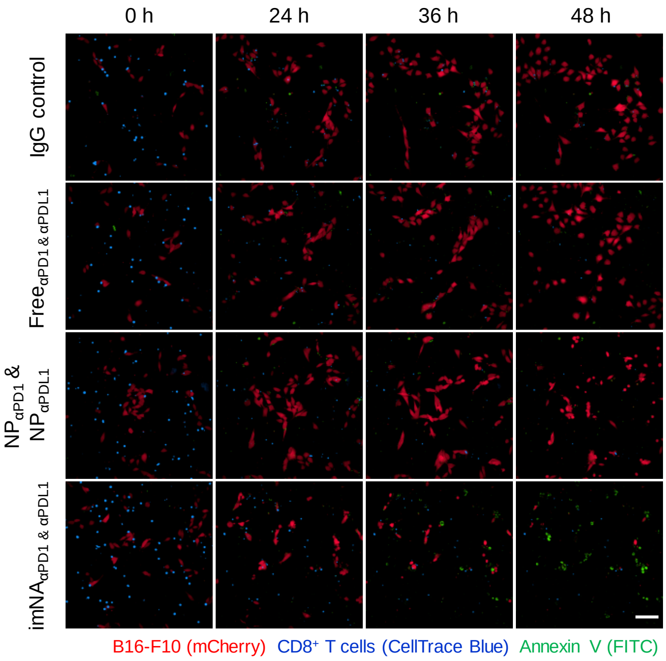

Supplementary Figure 11 | Real-time monitoring of T cell-mediated killing of tumor cells. B16-F10-mCherry cells were cocultured with CellTrace Blue labeled activated $CD8^+$ T cells at the cell ratio of 1:10, with the presence of IgG control, Free$_{\alpha PD1 \& \alpha PDL1}$, NP$_{\alpha PD1}$ & NP$_{\alpha PDL1}$, or imNA$_{\alpha PD1 \& \alpha PDL1}$. The concentration of IgG control and αPD1 & αPDL1 was 50 µg/mL. The process was monitored by a High Content Analysis System (Operetta CLS^{TM}, PerkinElmer), and images were obtained every 45 min for 48 h. Apoptotic cells were stained by FITC-conjugated-Annexin V. Scale bar = 50 µm.

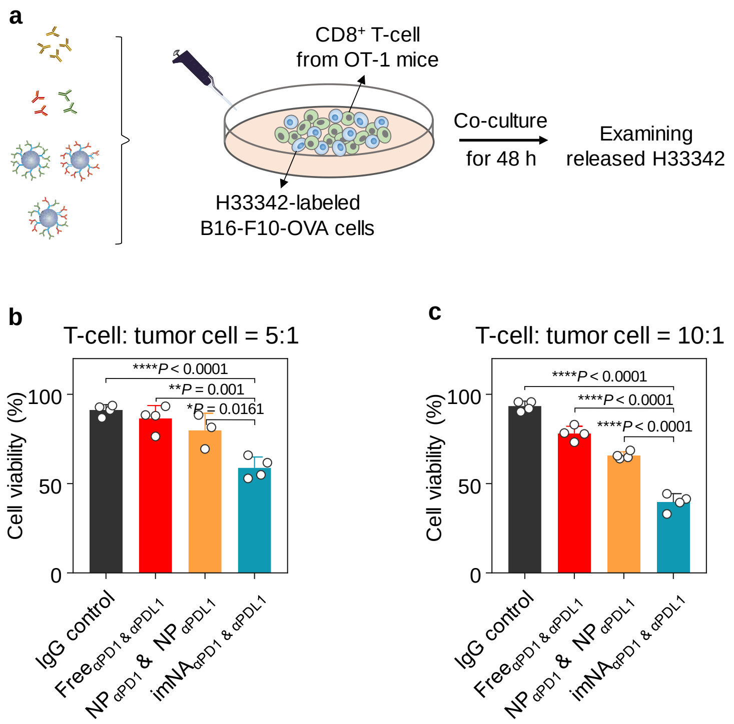

Supplementary Figure 12 | imNA$_{\alpha PD1\ \&\ \alpha PDL1}$ enhances the cytotoxicity of antigen-specific CD8^{+} T cells. a CD8^{+} T cells isolated from the spleen of OT-1 transgenic mice were stimulated with anti-CD3/CD28 antibodies and then incubated with Hoechst 33342-labeled B16-F10-OVA cells with the presence of different formulations. After 48 h incubation, the amount of Hoechst 33342 released by apoptotic tumor cells was examined using Infinite 200 PRO microplate plate reader and cell viabilies were calculated. Cell viability of B16-F10-OVA cells after 48 h incubation at the T cell: tumor cell ratios of 5:1 (**b**) and 10:1 (**c**). Data were presented as the mean ± s.d. n = 3-4 biologically independent samples, NP$_{\alpha PD1}$ & NP$_{\alpha PDL1}$ group in **b**: n = 3, other groups: n = 4. Statistical significance was calculated *via* one-way ANOVA with the Tukey post-hoc test. *P< 0.05, **P< 0.01, and ****P< 0.0001.

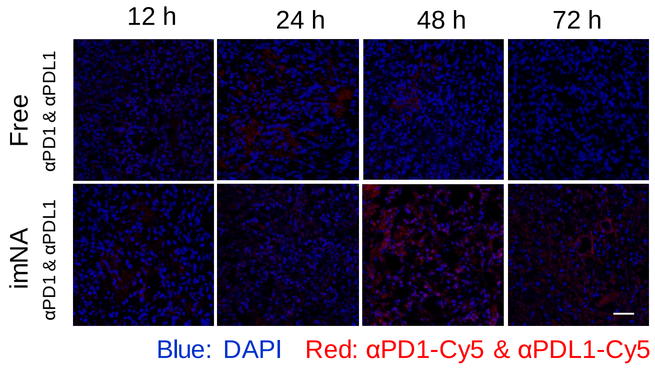

Supplementary Figure 13 | Immunofluorescence of mAbs distribution in tumor tissues with Free$_{\alpha PD1\ \&\ PDL1}$ or imNA$_{\alpha PD1\ \&\ \alpha PDL1}$ administration. Scale bars, 50 mm.

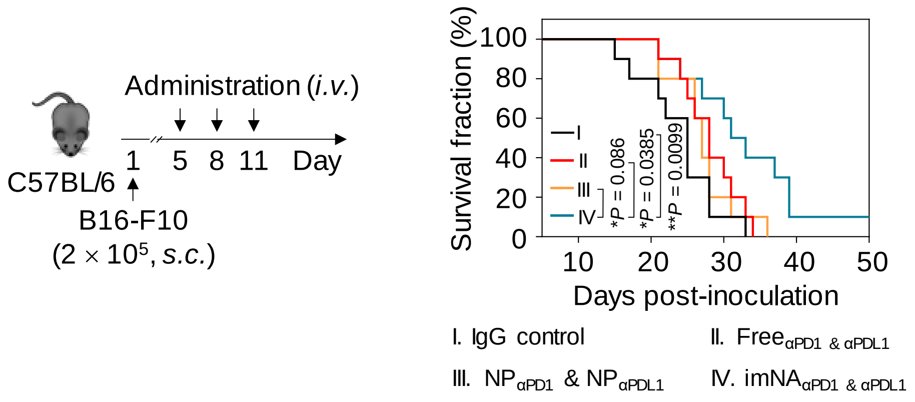

Supplementary Figure 14 | C57BL/6 mice bearing B16-F10 tumors were treated as indicated in **Figure 4d**, the survivals of treated mice were recorded. n = 10 biological independent mice. Survival curves were analyzed using a two-tailed log-rank (Mantel–Cox) test. *P< 0.05, **P< 0.01.

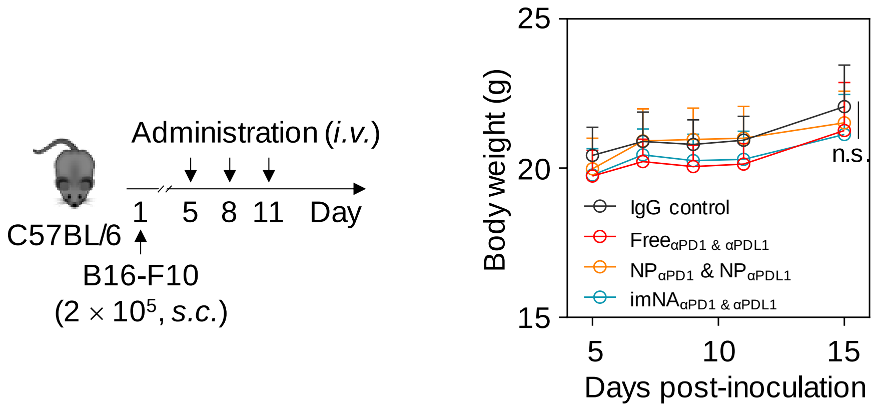

Supplementary Figure 15 | C57BL/6 mice bearing B16-F10 tumors were treated as indicated in **Figure 4d**, the body weight changes of C57BL/6 mice in the course of treatment. Data were presented as the mean ± s.d. n = 10 biological independent mice. Statistical significance was calculated *via* one-way ANOVA with a Tukey post-hoc test. n.s., no significant difference.

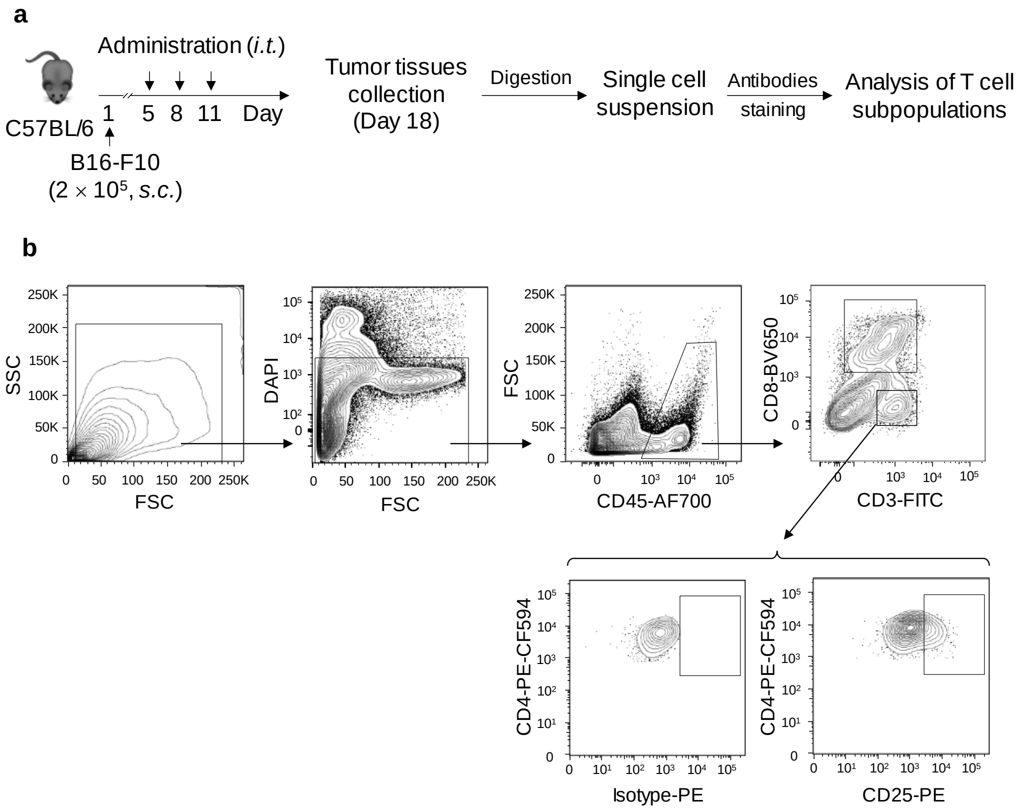

Supplementary Figure 16 | Gating strategy for analyzing T-lymphocyte subsets in B16-F10 melanoma tumors. a C57BL/6 bearing B16-F10 tumors were treated as indicated in **Figure 4d**, tumor tissues were collected and digested at the end of treatment, and the single-cell suspension was stained with antibody cocktail for flow cytometry examination. **b** Immune cell population was gated based on the expression of CD45, $CD45^+$ cells were further gated to determine $CD3^+CD8^+$, $CD4^+$ T cells, and $CD4^+CD25^+$ Treg cells. Cell populations were gated sequentially following arrows, and isotype controls were used for identifying the non-specific background.

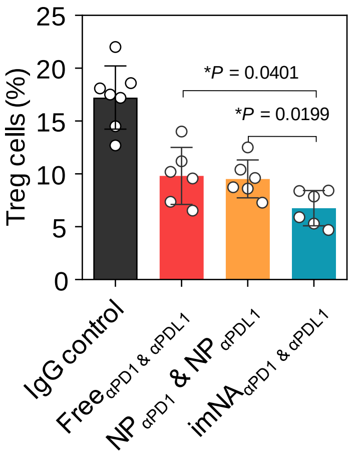

Supplementary Figure 17 | Percentage of $CD3^+CD4^+CD25^+$ regulatory T-cells (Treg) in B16-F10 melanoma tumors at the end of anti-tumor study. Data were presented as the mean ± s.d. n = 6-7 biologically independent mice, IgG control: n = 7, other groups: n = 6. Statistical significance was calculated *via* one-way ANOVA with a Tukey post-hoc test. *P< 0.05. This figure refers to **Figure 4d** and **Supplementary Figure 16**.

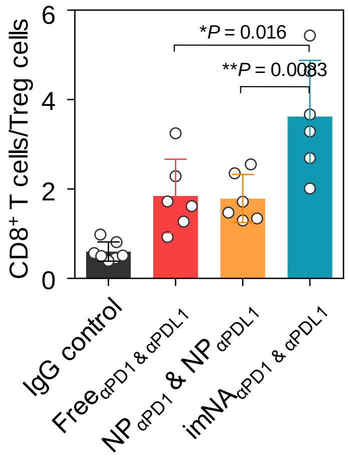

Supplementary Figure 18 | The ratios of CD8$^+$ T cells to Treg cells in B16-F10 melanoma tumors at the end of anti-tumor study. Data were presented as the mean ± s.d. n = 6-7 biologically independent mice, IgG control: n = 7, other groups: n = 6. Statistical significance was calculated *via* one-way ANOVA with a Tukey post-hoc test. **P*< 0.05, ***P*< 0.01. This figure refers to **Figure 4d** and **Supplementary Figure 16**.

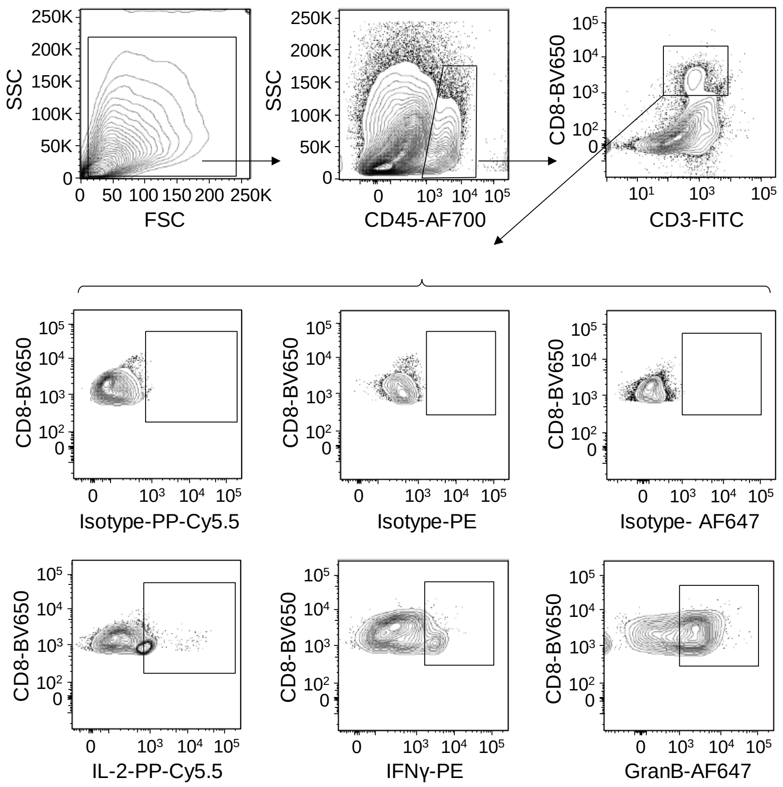

Supplementary Figure 19 | Gating strategy for analyzing subsets of cytokine-secreting CD8$^+$ T cells. C57BL/6 bearing B16-F10 tumors were treated as indicated in **Figure 4d**, tumor tissues were collected and digested at the end of treatment, intracellular cytokines in tumor-infiltrating lymphocytes were stained and examined by flow cytometry. Immune cell population was gated based on the expression of CD45, CD45$^+$ cells were further gated to determine CD3$^+$CD8$^+$IL2$^+$, IFN-γ$^+$ or GranB$^+$ cells. Isotype controls were used for identifying the non-specific background in flow cytometric analysis.

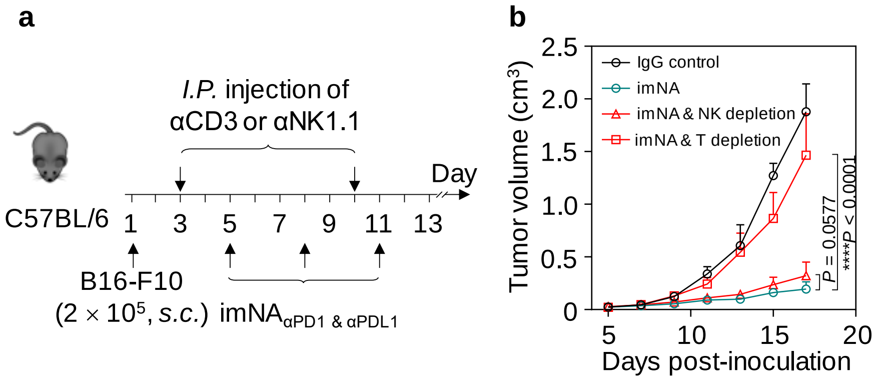

Supplementary Figure 20 | T cells play a predominant role in imNA$_{\alpha PD1\ \&\ \alpha PDL1}$-mediated antitumor effect. a Subcutaneous B16-F10 tumor model was established. T cells and NK cells were depleted *via* intraperitoneal injection of 200 μg mAb to CD3 or NK1.1 three days post tumor inoculation, and antibodies were injected weekly for two times. imNA$_{\alpha PD1\ \&\ \alpha PDL1}$ was administrated every three days for three repeats since 5th days post tumor inoculation every, the injection dose of αPD1 and αPDL1 was 2.5 mg/kg. **b** Tumor growth of B16-F10 tumors after treated with imNA$_{\alpha PD1\ \&\ \alpha PDL1}$ with or without T cells or NK cells depletion. Data were presented as the mean ± s.d. n = 6 biologically independent mice. Statistical significance was calculated *via* one-way ANOVA with a Tukey post-hoc test. *$P < 0.05$; ****$P < 0.0001$.

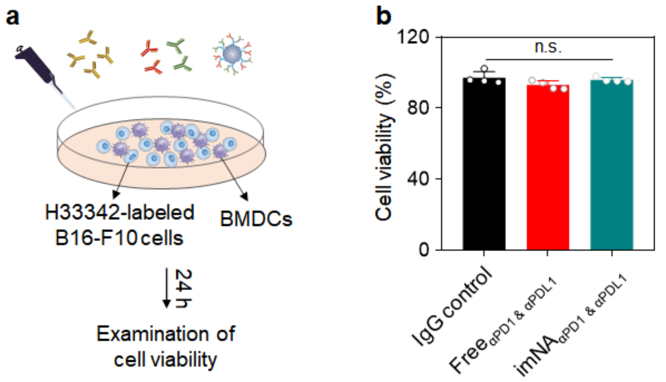

Supplementary Figure 21 | imNA$_{\alpha PD1\ \&\ \alpha PDL1}$ do not enhance the cytotoxicity of BMDCs against tumor cells. a BMDCs isolated from bone marrow were cocultured with Hoechst 33342-labeled B16-F10 with the presence of Free$_{\alpha PD1\ \&\ \alpha PDL1}$ or imNA$_{\alpha PD1\ \&\ \alpha PDL1}$, the concentration of αPD1 & αPDL1 was 10 μg/mL. and the tumor cell: BMDCs ratio is 1:1. After 48 h incubation, the amount of Hoechst 33342 released by apoptotic tumor cells was examined using Infinite 200 PRO microplate plate reader and cell viabilities were calculated. **b** Cell viability of B16-F10 cells after 24 h incubation. Data were presented as the mean ± s.d. n = 4 biologically independent samples. Statistical significance was calculated *via* one-way ANOVA with the Tukey post-hoc test. n.s., no significant difference.

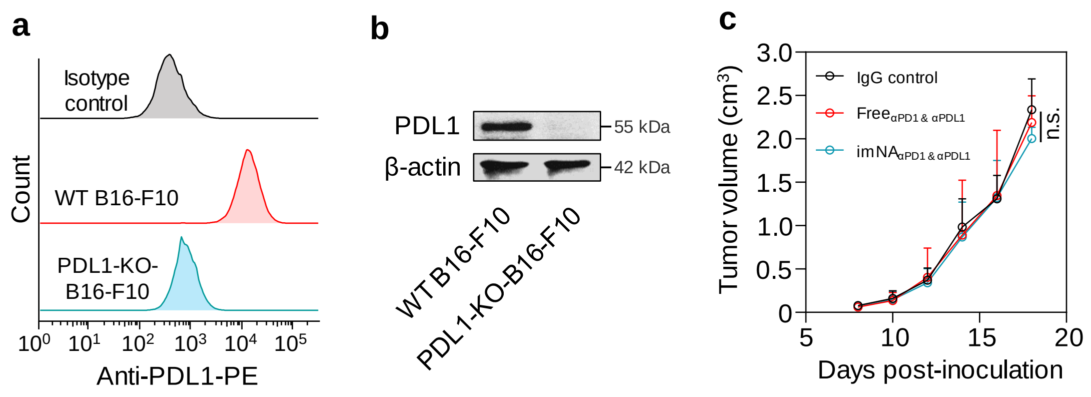

Supplementary Figure 22 | imNA$_{\alpha PD1\ \&\ \alpha PDL1}$ exhibited marginal benefits in terms of tumor control in PDL1-KO-B16-F10 model. To generate PDL1-KO-B16-F10 cells, wide-type (WT) B16-F10 cells were transfected with plasmid DNA (pX330/sgPD-L1) encoding Cas9 protein and single-guide RNA targeting PD-L1. Seven days after transfection, cells were stimulated with IFN-γ (20 ng/mL) for 48 h to upregulate PDL1 on WT cells and stained with PE-labeled PDL1 antibody for FACS-sorting of PDL1-KO cells. PD-L1 expression in sorted cells was examined by flow cytometry (**a**) and Western blotting (**b**). The dilutions for antibodies were made according to the manufacturer recommendations. (**c**) Tumor growth of mice receiving imNA$_{\alpha PD1\ \&\ \alpha PDL1}$ treatment. The subcutaneous PDL1-KO-B16-F10 model was established in C57BL/6 mice, and when tumor volume reached 50 mm^3, tumor-bearing mice were treated with IgG control, Free$_{\alpha PD1\ \&\ \alpha PDL1}$ or imNA$_{\alpha PD1\ \&\ \alpha PDL1}$ following a q3dx3 course (three times at intervals of three days). The data are presented as means ± s.d. n = 6 biological independent mice. Statistical significance was calculated *via* one-way ANOVA with the Tukey post-hoc test. n.s., no significant difference.

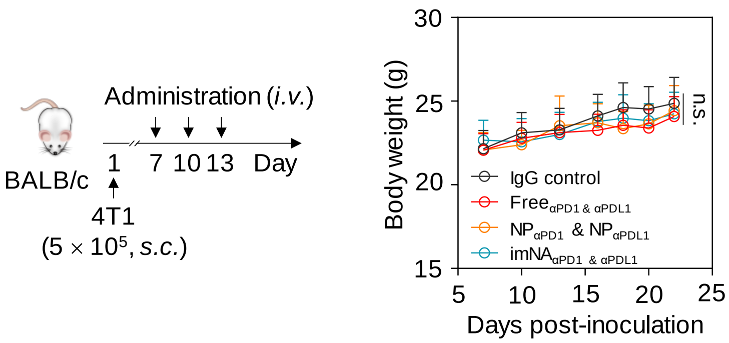

Supplementary Figure 23 | BALB/c mice bearing 4T1 tumors were treated as indicated in **Figure 4k**, and the body weight changes in the course of treatment. Data were presented as the mean ± s.d. n = 10 biological independent mice. Statistical significance was calculated *via* one-way ANOVA with the Tukey post-hoc test. n.s., no significant difference.

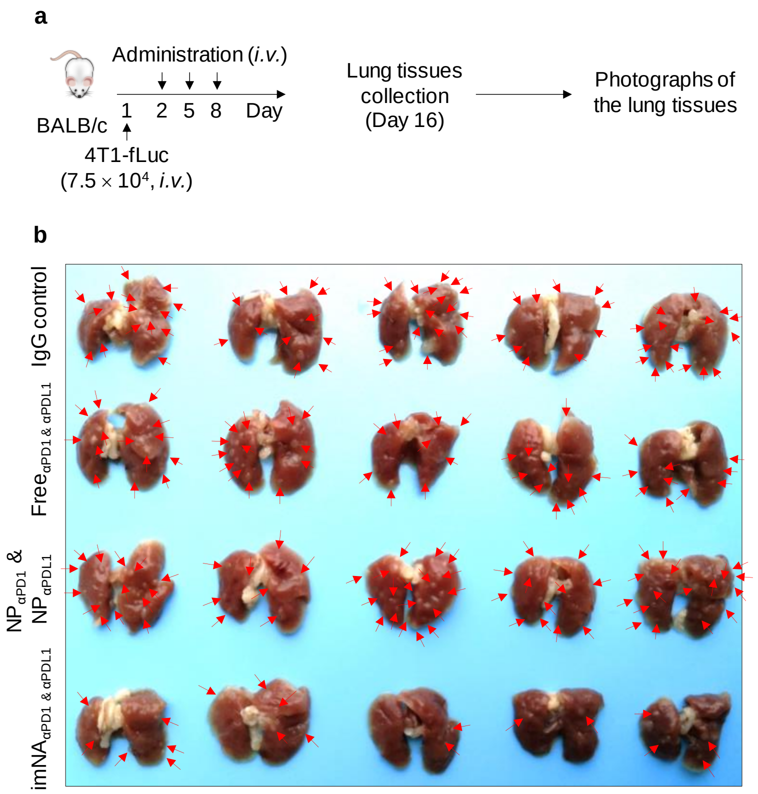

Supplementary Figure 24 | imNA$_{\alpha PD1\ \&\ \alpha PDL1}$ could eliminate circulating tumor cells and inhibit tumor metastasis. a Mice were intravenously injected with 4T1 cells expressing firefly luciferase (4T1-fLuc) and then received IgG control, Free$_{\alpha PD1\ \&\ \alpha PDL1}$, NP$_{\alpha PD1}$ & NP$_{\alpha PDL1}$ or imNA$_{\alpha PD1\ \&\ \alpha PDL1}$ following a q3dx3 course from the second day. **b** Lung tissues were collected on 16 days post-infusion and photographed; red arrows indicated tumor nodules on lung tissue.

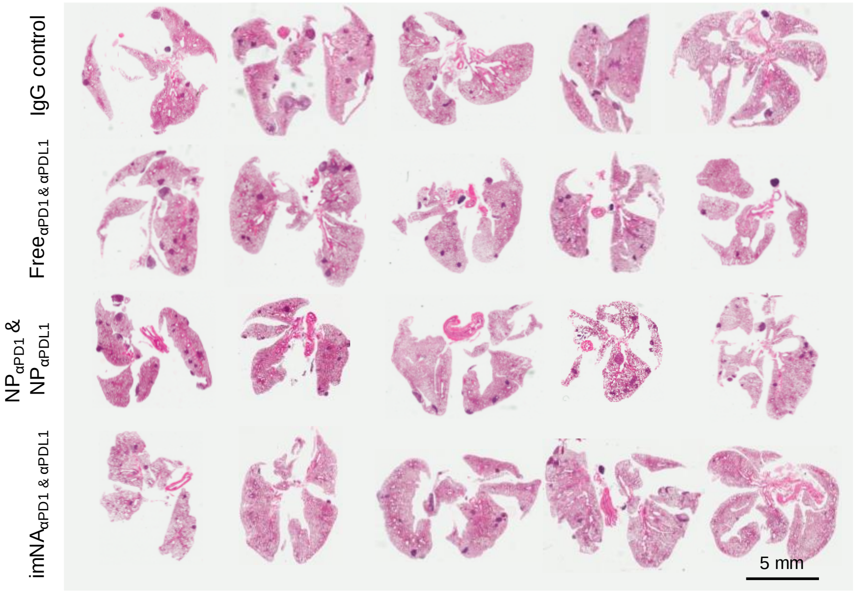

Supplementary Figure 25 | H&E staining of the lung tissue collected at day 16. Scale bar = 5 mm. Treatment schedule was indicated in **Supplementary Figure 24**.

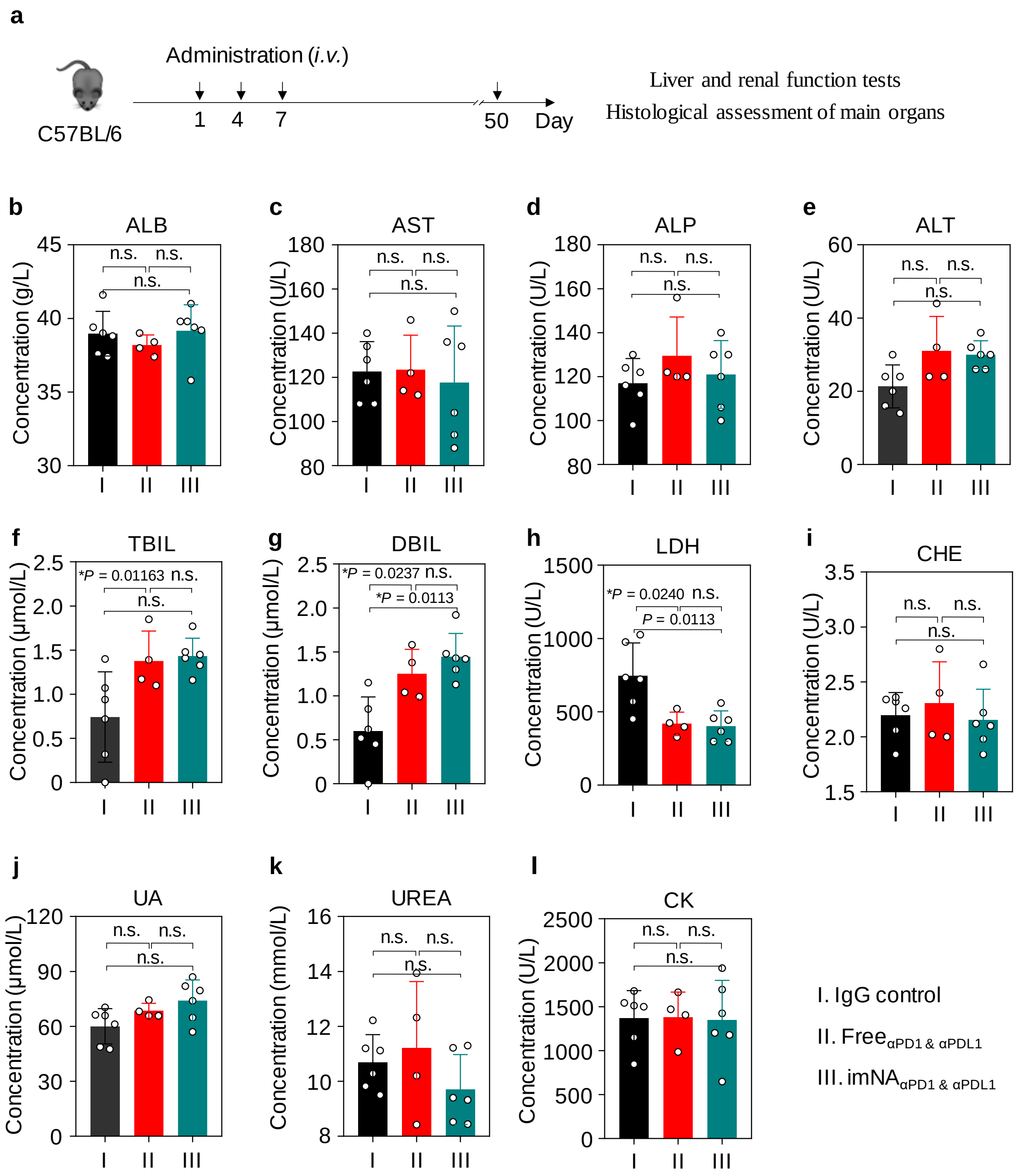

Supplementary Figure 26 | Liver and renal function tests. a Male C57BL/6 mice (6-8 weeks old) were intravenously injected with Free$_{\alpha PD1\ \&\ \alpha PDL1}$ or imNA$_{\alpha PD1\ \&\ \alpha PDL1}$ (5.0 mg/kg) every three days for three repeats. Six weeks post last injection, serum samples and main organs were harvested for examination. Concentrations of ALB (**b**), AST (**c**), ALP (**d**), ALT (**e**), TBIL (**f**), DBIL (**g**), LDH (**h**), CHE (**i**), UA (**j**), UREA (**k**) and CK (**l**) in serum were measured. Data were presented as the mean ± s.d., n = 4-6 biologically independent mice (IgG control: n = 6, Free$_{\alpha PD1\ \&\ \alpha PDL1}$: n = 4, imNA$_{\alpha PD1\ \&\ \alpha PDL1}$: n = 6). Statistical significance was calculated *via* one-way ANOVA with a Tukey post-hoc test. *$P <$ 0.05. ns, no significant difference.

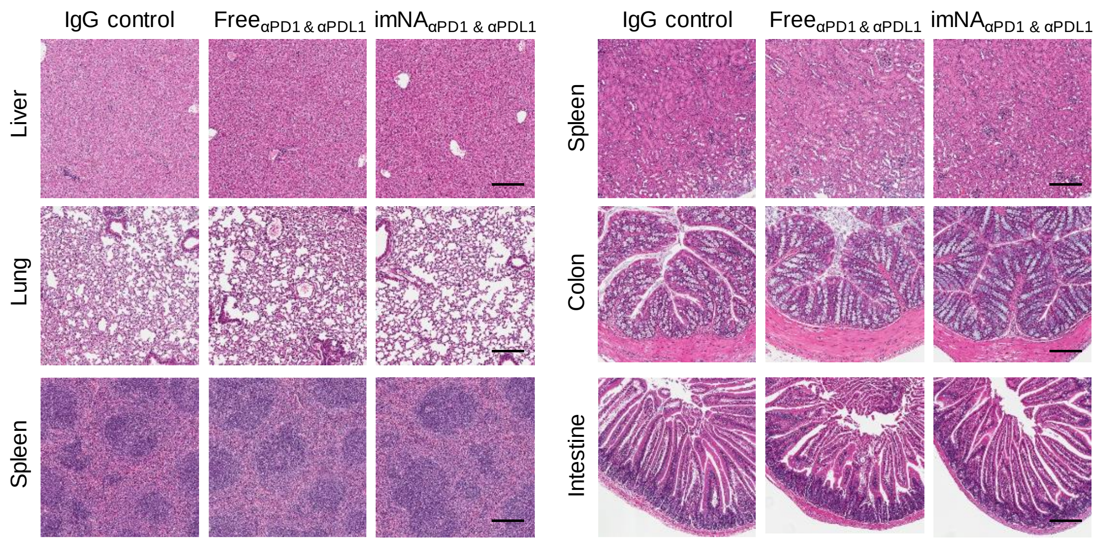

Supplementary Figure 27 | Histological assessment of liver, lung, spleen, kidney, colon and intestine. Scale bar, 200 μm. This figure refers to **Supplementary Figure 26**.

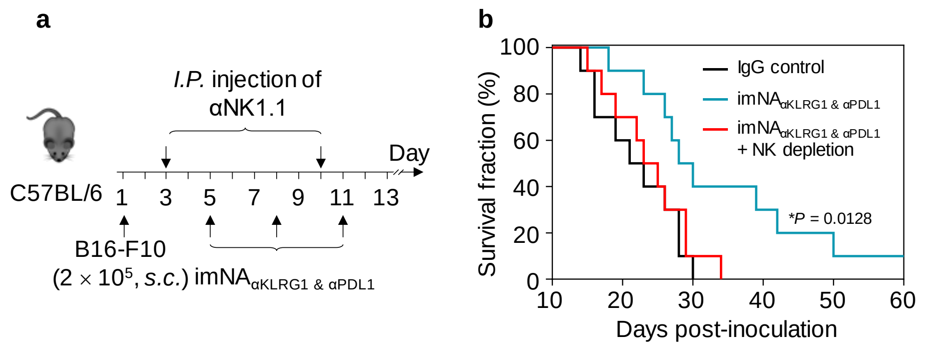

Supplementary Figure 28 | a Subcutaneous B16-F10 tumor model was established and mice were intraperitoneal injected with anti-NK1.1 antibody three days post tumor inoculation for NK cell depletion. **b** Survival curves of the mice receiving imNA$_{\alpha KLRG1\ \&\ \alpha PDL1}$ or imNA$_{\alpha KLRG1\ \&\ \alpha PDL1}$ plus depletion of NK cells. n = 10 biological independent mice. Survival curves were analyzed using a two-tailed log-rank (Mantel–Cox) test, $*P < 0.05$.

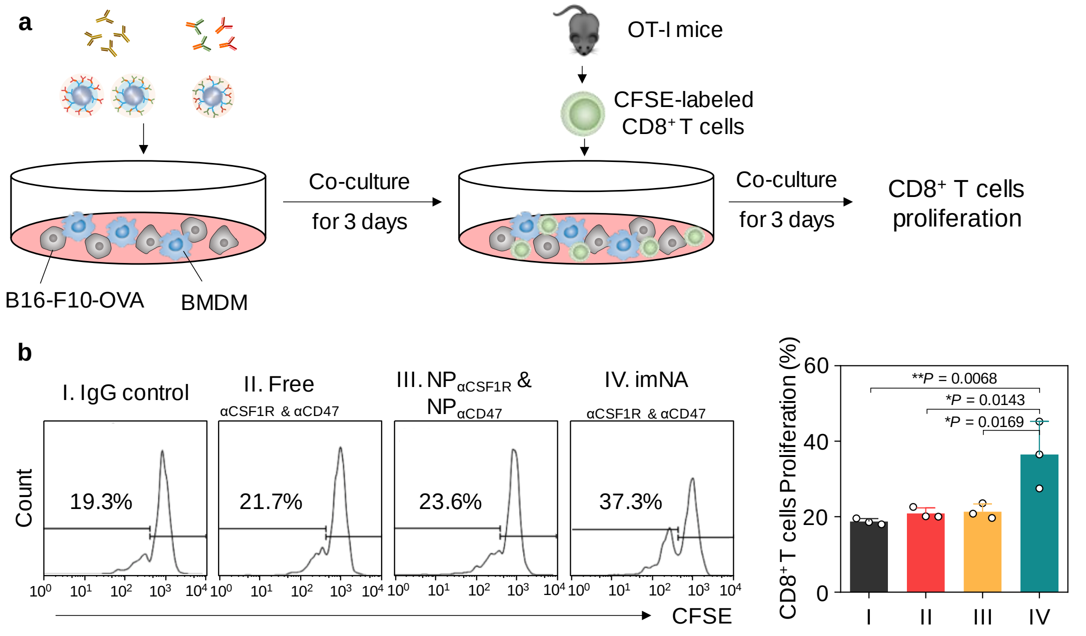

Supplementary Figure 29 | Macrophages prime CD8$^+$ T cells to proliferate after phagocytosis of cancer cells with the assistance of imNA$_{\alpha CSF1R\ \&\ \alpha CD47}$. **a** Experimental protocol. B16-F10-OVA and BMDMs were cocultured with the presence of IgG control, Free$_{\alpha CSF1R\ \&\ \alpha CD47}$, NP$_{\alpha CSF1R}$ & NP$_{\alpha CD47}$ or imNA$_{\alpha CSF1R\ \&\ \alpha CD47}$ for three days. CFSE-labeled CD8$^+$ T cells (OT-I) were then cocultured with treated BMDMs for three days, followed by CFSE-dilution assay. **b** Representative histograms of CFSE staining in CD8$^+$ cells from different groups. **c** Percentages of proliferating CD8$^+$ T cells. Data were presented as the mean ± s.d. n = 3 biologically independent samples. Statistical significance was calculated *via* one-way ANOVA with a Tukey post-hoc test. *$P < 0.05$; **$P < 0.01$.

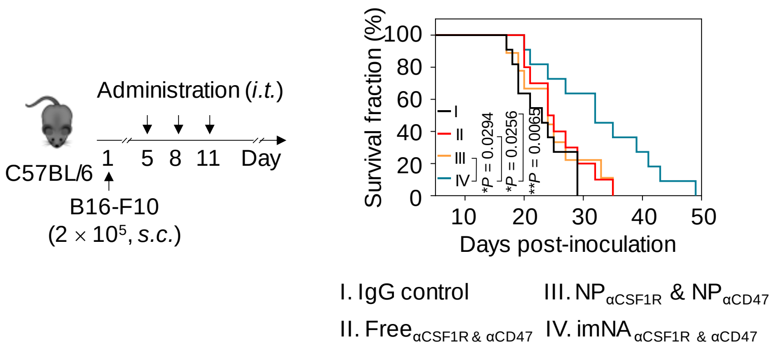

Supplementary Figure 30 | C57BL/6 mice bearing B16-F10 tumors were treated as indicated in **Figure 5i**, the survivals of treated mice were recorded. n = 10 biological independent mice. Survival curves were analyzed using a two-tailed log-rank (Mantel–Cox) test. *P< 0.05, **P< 0.01.

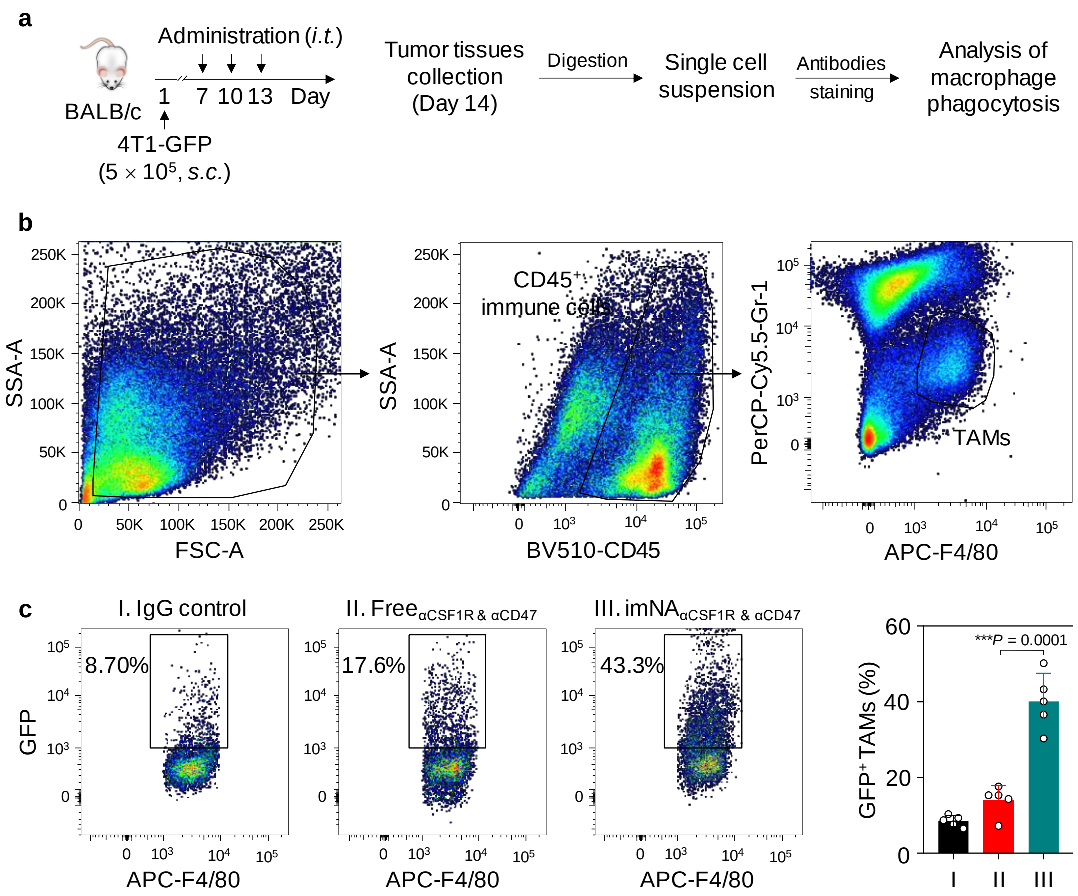

Supplementary Figure 31 | imNA$_{\alpha CSF1R\ \&\ \alpha CD47}$ promotes phagocytosis of GFP-expressing 4T1 cells by tumor-associated macrophages (TAMs) *in vivo*. **a** Mice bearing orthotopic 4T1-GFP breast tumors were treated with IgG control, Free$_{\alpha CSF1R\ \&\ \alpha CD47}$, or imNA$_{\alpha CSF1R\ \&\ \alpha CD47}$ every three days for three times when the tumor volumes reached about 50 mm^3 (the equivalent dose of αCSF1R and αCD47 was 1.5 mg/kg, and the dose of IgG control was 3.0 mg/kg). Tumor tissues were collected for digestion 24 h post the last injection. Macrophages were stained and the macrophage phagocytosis against 4T1-GFP was examined by flow cytometry. **b** Tumor tissues were collected and digested at the end of treatment, tumor-infiltrating immune cells were stained with antibody cocktails, and TAMs were gated as CD45$^+$CD11b$^+$Gr-1$^-$F4/80$^+$. **c** The phagocytosis of tumor cells by TAMs was evaluated by examining the percentage of GFP$^+$ TAMs. Data were presented as the mean ± s.d. n = 5 biological independent mice. Statistical significance was calculated *via* one-way ANOVA with a Tukey post-hoc test. ***P< 0.001.

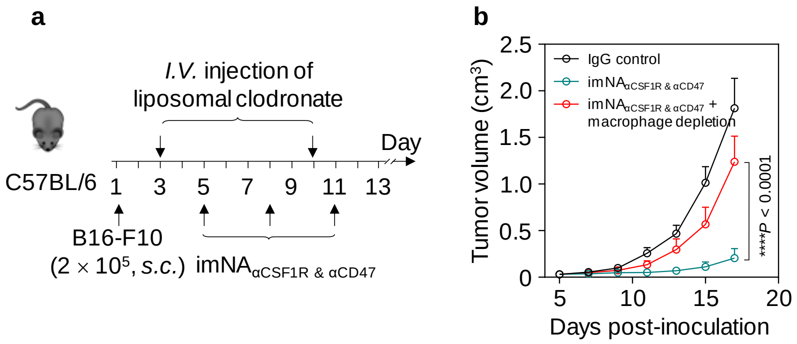

Supplementary Figure 32 | a Subcutaneous B16-F10 tumor model was established and mice were intravenously injected with 1 mg liposomal clodronate (ClodronateLiposomes.org, Netherlands) three days post tumor inoculation for macrophage depletion. Liposomal clodronate was injected weekly for two time. **b** Tumor growth curve of B16-F10 tumor-bearing mice receiving $imNA_{\alpha CSF1R\ \&\ \alpha CD47}$ or $imNA_{\alpha CSF1R\ \&\ \alpha CD47}$ plus depletion of macrophages. Data were presented as the mean ± s.d. n = 6 biological independent mice. Statistical significance was calculated *via* one-way ANOVA with a Tukey post-hoc test. $^{****}P < 0.0001$.

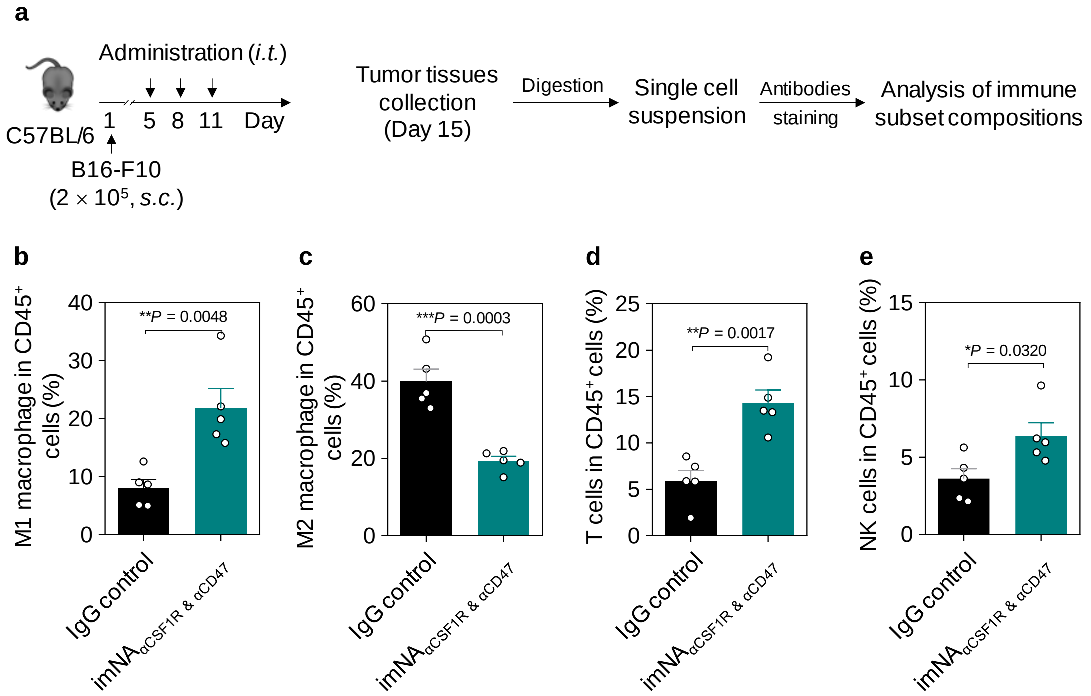

Supplementary Figure 33 | imNA$_{\alpha CSF1R\ \&\ \alpha CD47}$ reverses the immunosuppressive tumor microenvironment. a Subcutaneous B16-F10 tumor model was established and tumor-bearing mice were treated with IgG control antibody or imNA$_{\alpha CSF1R\ \&\ \alpha CD47}$. At the end of the treatment, tumor tissues were collected for digestion, the single-cell suspension was stained with antibody cocktails for examining the immune subset compositions, including M1 macrophages (**b**), M2 macrophages (**c**), T cells (**d**), and NK cells (**e**). Data were presented as the mean ± s.d. n = 5 biological independent mice. Statistical significance was calculated via one-way ANOVA with a Tukey post-hoc test. *$P < 0.05$; **$P < 0.01$, ***$P < 0.001$.

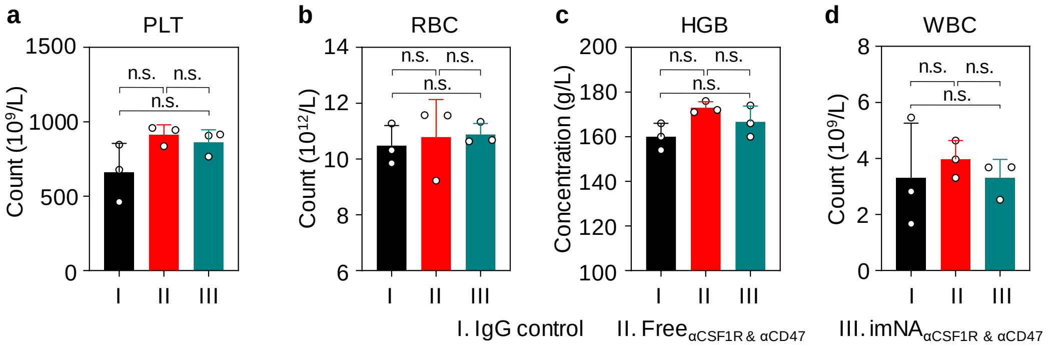

Supplementary Figure 34 | imNA$_{αCSF1R \& αCD47}$ administration does not induce anemia. Male C57BL/6 mice (6-8 weeks old) were intravenously injected with Free$_{αCSF1R \& αCD47}$ or imNA$_{αCSF1R \& αCD47}$ every three days for three circles. The injection dose of αCSF1R and αCD47 was 1.5 mg per kg mouse body weight. Platelet, PLT (**a**), red blood cell, RBC (**b**), hemoglobin, HGB (**c**) and white blood cell, WBC (**d**) in the peripheral blood are measured. Data were presented as the mean ± s.d. n = 3 biological independent mice. Statistical significance was calculated *via* one-way ANOVA with a Tukey post-hoc test. n.s., no significant difference.
