# Supplementary material for: Immunomodulating nano-adaptors potentiate antibody-based cancer immunotherapy
Source: Nat Commun. 2021 Mar 1;12:1359. doi: 10.1038/s41467-021-21497-6 (PMC7921676; doi:10.1038/s41467-021-21497-6)
Supplement: Supplementary file 2 — Description of Additional Supplementary Files [file 41467_2021_21497_MOESM2_ESM.docx]

**Description of Additional Supplementary Files**

File Name: **Supplementary Movie 1**

Description: **The dynamic process of T cell-mediated tumor cell killing in co-cultured cells treated with IgG control.** To detect the dynamic process of cell apoptosis, 5.0 × 10^3^ B16-F10-mCherry cells and 5.0 × 10^4^ stimulated CD8+ T cells labeled with CellTrace Blue were seeded into CellCarrierUltra ULA 96-well microplates (PerkinElmer). Co-cultured cells were treated with IgG control (20 μg/mL). FITC conjugated recombinant Annexin V (Annexin V-FITC, Thermo Fisher Scientific) was added to the medium (final concentration was 1 μg/mL) to detect apoptotic cells. Images of co-cultured cells were continuously acquired every 45 min using the Operetta CLS™ High-Content Analysis System (PerkinElmer) for 48 h and were processed into a dynamic process.

File Name: **Supplementary Movie 2**

Description: **The dynamic process of T cell-mediated tumor cell killing in co-cultured cells treated with Free_αPD1 & αPDL1_.** To detect the dynamic process of cell apoptosis, 5.0 × 10^3^ B16-F10-mCherry cells and 5.0 × 10^4^ stimulated CD8+ T cells labeled with CellTrace Blue were seeded into CellCarrierUltra ULA 96-well microplates (PerkinElmer). Co-cultured cells were treated with Free_αPD1 & αPDL1_; the concentration of αPD1 and αPDL1 was 10 μg/mL. FITC conjugated recombinant Annexin V (Annexin V-FITC, Thermo Fisher Scientific) was added to the medium (final concentration was 1 μg/mL) to detect apoptotic cells. Images of co-cultured cells were continuously acquired every 45 min using the Operetta CLS™ High-Content Analysis System (PerkinElmer) for 48 h and were processed into a dynamic process.

File Name: **Supplementary Movie 3**

Description: **The dynamic process of T cell-mediated tumor cell killing in co-cultured cells treated with NP_αPD1_ & NP_αPDL1_.** To detect the dynamic process of cell apoptosis, 5.0 × 10^3^ B16-F10-mCherry cells and 5.0 × 10^4^ stimulated CD8+ T cells labeled with CellTrace Blue were seeded into CellCarrierUltra ULA 96-well microplates (PerkinElmer). Co-cultured cells were treated with NP_αPD1_ & NP_αPDL1_. The concentration of αPD1 and αPDL1 was 10 μg/mL. FITC conjugated recombinant Annexin V (Annexin V-FITC, Thermo Fisher Scientific) was added to the medium (final concentration was 1 μg/mL) to detect apoptotic cells. Images of co-cultured cells were continuously acquired every 45 min using the Operetta CLS™ High-Content Analysis System (PerkinElmer) for 48 h and were processed into a dynamic process.

File Name: **Supplementary Movie 4**

Description: **The dynamic process of T cell-mediated tumor cell killing in co-cultured cells treated with imNA_αPD1 & αPDL1_.** To detect the dynamic process of cell apoptosis, 5.0 × 10^3^ B16-F10-mCherry cells and 5.0 × 10^4^ stimulated CD8+ T cells labeled with CellTrace Blue were seeded into CellCarrierUltra ULA 96-well microplates (PerkinElmer). Co-cultured cells were treated with imNA_αPD1 & αPDL1_; the concentration of αPD1 and αPDL1 was 10 μg/mL. FITC conjugated recombinant Annexin V (Annexin V-FITC, Thermo Fisher Scientific) was added to the medium (final concentration was 1 μg/mL) to detect apoptotic cells. Images of cocultured cells were continuously acquired every 45 min using the Operetta CLS™ High-Content Analysis System (PerkinElmer) for 48 h and were processed into a dynamic process.
